# Lipid Dyshomeostasis and Inherited Cerebellar Ataxia

**DOI:** 10.1007/s12035-022-02826-2

**Published:** 2022-04-14

**Authors:** Jin Zhao, Huan Zhang, Xueyu Fan, Xue Yu, Jisen Huai

**Affiliations:** 1 The Second Affiliated Hospital of Xinxiang Medical University (Henan Mental Hospital), Xinxiang, 453000 China; 2 Institute of Psychiatry and Neuroscience, Xinxiang Medical University, Xinxiang, 453003 China

**Keywords:** Cerebellar ataxia, Lipid homeostasis, Common signaling pathway, Diagnosis, Treatment

## Abstract

Cerebellar ataxia is a form of ataxia that originates from dysfunction of the cerebellum, but may involve additional neurological tissues. Its clinical symptoms are mainly characterized by the absence of voluntary muscle coordination and loss of control of movement with varying manifestations due to differences in severity, in the site of cerebellar damage and in the involvement of extracerebellar tissues. Cerebellar ataxia may be sporadic, acquired, and hereditary. Hereditary ataxia accounts for the majority of cases. Hereditary ataxia has been tentatively divided into several subtypes by scientists in the field, and nearly all of them remain incurable. This is mainly because the detailed mechanisms of these cerebellar disorders are incompletely understood. To precisely diagnose and treat these diseases, studies on their molecular mechanisms have been conducted extensively in the past. Accumulating evidence has demonstrated that some common pathogenic mechanisms exist within each subtype of inherited ataxia. However, no reports have indicated whether there is a common mechanism among the different subtypes of inherited cerebellar ataxia. In this review, we summarize the available references and databases on neurological disorders characterized by cerebellar ataxia and show that a subset of genes involved in lipid homeostasis form a new group that may cause ataxic disorders through a common mechanism. This common signaling pathway can provide a valuable reference for future diagnosis and treatment of ataxic disorders.

**Supplementary Information:**

The online version contains supplementary material available at 10.1007/s12035-022-02826-2.

## Introduction

Ataxia is a neurological disorder characterized by clinical abnormalities of balance, gait, extremity, eye movement, and impaired speech due to degeneration of the cerebellum and its connections [1–4]. It may be divided into three types: sporadic, acquired, and inherited ataxias [4–6]. Inherited ataxias are further subdivided into autosomal dominant cerebellar ataxias (ADCAs/SCAs), autosomal recessive cerebellar ataxias (ARCAs), and X-linked cerebellar ataxias (XLCAs) [7–11]. The inherited ataxias are genetically diverse; thus far, 47 subtypes of ADCAs/SCAs [12–14], 59 subtypes of ARCAs [15], and more than 20 subtypes of XLCAs [11] have been identified. Moreover, approximately one-third of patients with clinical suspicion of ADCAs/SCAs and 50% of ARCAs remain without a molecular diagnosis [16–18].

Regarding the pathological processes of different types of inherited ataxias, ADCAs mostly affect the cerebellum, brainstem, and spinal cord [2],while ARCAs and XLCAs involve both the central and peripheral nervous systems, and non-neurological systems in some cases [11, 18]. Based on shared molecular mechanisms, ADCAs/SCAs are mainly categorized into four groups (namely, the CAG repeat–polyglutamine ataxias, ataxias associated with ion channel dysfunction, ataxias associated with mutations in signal transduction molecules, and ataxias associated with noncoding repeats) [19, 20]. However, ARCAs and XLCAs are categorized into five and two groups, respectively [2, 11, 21]. Of note, due to the considerable variety and salient overlap of clinical features among different disorders, rational classification has been impeded [7, 8, 15, 22]. In particular, some ADCAs/SCAs are not significantly different from ARCAs, named autosomal recessive spinocerebellar ataxias (SCARs) [23]**.**

For many years, cerebellar ataxia has been thought to be incurable, and the treatment options are mainly limited to managing the symptoms, rather than treating the direct cause of the diseases [12, 24, 25]. However, as a result of intensive studies involving the molecular mechanisms, several promising therapeutic strategies have been developed [26]. In particular, antisense oligonucleotides (ASOs) that target the polyQ-coding SCA genes are under development and have been used in preclinical animal models [27–30]. Clinical trials of ASOs in SCA patients have been planned [12]. In addition, targeting the deranged calcium signaling pathway has been proposed as another potential therapeutic strategy for the different types of ADCAs/SCAs, and preclinical experiments have obtained some promising results [31–34].

**Fig. 1:**
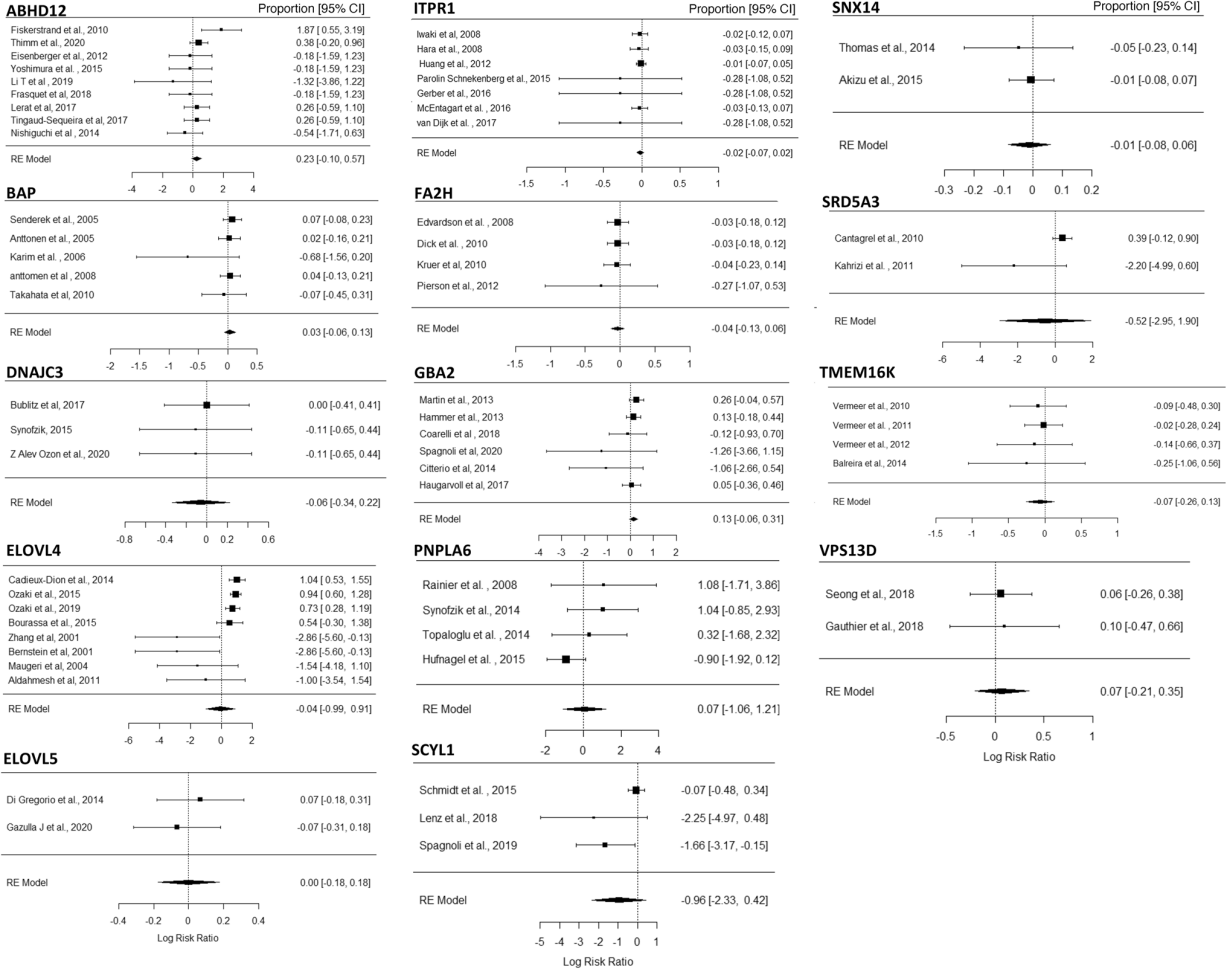
Forest plot for frequency of ataxia related to genes involved in lipid homeostasis. We have that found 18 molecules related to lipid homeostasis are probably linked to cerebellar ataxia. We selected 14 of these for meta-analysis. However, regarding the remaining four genes, there are only animal cases available or the data is insufficient for analysis. Our meta-analysis results indicate that all 14 genes show high risk ratios for ataxia. The Random Effect (RE) model measures the mean weighted effect size (indicated by diamonds) and Confidence Interval (CI). Each square size reflects the study weight

Although some important advances have been made, the pathogenesis of ataxia caused by many genes is still not fully understood [10, 19]. Accumulating evidence indicates that other common pathogenic mechanisms exist within each type of inherited ataxia [35–38]. For example, the emerging common pathways underlying ARCAs include three main clusters, namely mitochondrial dysfunction, impaired DNA repair, and complex lipid homeostasis [35]. Based on these new findings, we speculate that there may be a common mechanism for different types of disorders with cerebellar ataxia as hallmarks. However, to date, there have been no reports of this. Recently, we systematically and comprehensively reviewed the PubMed literature and screened the OMIM and GeneReviews databases. In the OMIM databank, when entering “cerebellar ataxia’’ as the search keyword, more than three thousand entries appear. We checked each entry individually and in combination with screening GeneReviews. We finally determined that more than 300 genes were closely associated with cerebellar ataxia, not including those whose defects are related to ataxia, but there is no clear evidence showing that their defects cause cerebellar disorders. We then sorted these genes and found that a group of genes involved in maintaining lipid homeostasis are linked to different types of cerebellar ataxia, including genes that are linked to ARCAs and have been reviewed by Synofzik et al. [35]. Since members of this gene set are not only linked to ARCAs, as mentioned above, but also to other types of cerebellar ataxia, we speculate that they may represent a common mechanism of disorders characterized by cerebellar ataxia. Thus, in this article, the literature on this gene set is reviewed to reveal their internal connections to cerebellar ataxia. Notably, some genes that have been reviewed by Synofzik et al. are also included, as new discoveries have been reported and/or more detail is necessary. We extracted the numbers of patients with and without clinical ataxic symptoms from tables, texts, and figures from references regarding the genes, and transferred these to a master MS Excel spread sheet (Table S1) for subsequent meta-analysis using “metafor’’ (R package) [39]. Our analyses indicate that all analyzed genes show high risk ratios for ataxia (Fig. 1). It should be mentioned that this analysis is subject to many confounders which have not been taken into account. Interestingly, most proteins encoded by the genes reviewed in this manuscript are located in the endoplasmic reticulum (ER), which is an important organelle of lipid metabolism. In subsequent sections of this review, we have summarized the latest physiological and pathological functions of these proteins, as well as their intrinsic mechanisms leading to ataxia. Our goal is to determine the potential common mechanism underlying different ataxias and evaluate the possibility of a common signaling pathway as potential targets for future precision treatments.

## Genes Involved in Lipogenesis or Lipolysis

ER-localized enzymes catalyze the synthesis of most cellular membrane lipids. These enzymes also drive carbohydrates and nutritionally derived lipids into storage lipids to maintain lipid homeostasis [40]. The vast majority of lipids synthesized in the ER include phospholipids, triacylglycerols (TAGs), cholesterols, and cholesteryl esters [41, 42]. Sphingolipid synthesis starts in the ER until the formation of ceramide, and is completed in the Golgi apparatus, where complex lipids are synthesized [43–45]. Besides being components of the cell membrane, these lipids also play vital roles in many biological processes [46, 47]. Therefore, both synthesis and decomposition of lipids must be finely regulated [48, 49].

### Patatin-Like Phospholipase Domain-Containing Protein 6 (PNPLA6)

Phospholipids make up the bilayer membrane matrix, and their composition and complexity in various membranes of eukaryotic cells differ [50, 51]. The most abundant lipid in the ER membrane is phosphatidylcholine (PC) [51, 52]. In eukaryotic cells, PC is synthesized through the Kennedy pathway [53, 54] or another pathway that catalyzes the conversion of PE to PC [55–58]. In contrast, PNPLA6 is responsible for the degradation of PC into glycerophosphocholine [59–61]. In addition, PNPLA6 and its *Drosophila* ortholog can also hydrolyze lysophosphatidylcholine (LPC) [62–64]. PNPLA6 is localized in the ER membrane [60, 65]. It is highly expressed in cerebellar Purkinje cells and its deficiency causes reduced dendritic trees and loss of Purkinje cells [66–68]. Mutations in PNPLA6 are associated with a spectrum of neurodegenerative disorders, that present clinical features ranging from pure cerebellar ataxia to complex forms of ataxia associated with other symptoms [68–74].

Thus far, most pathogenic mutations in PNPLA6 were observed in its catalytic or regulatory domain [62, 71, 75]. These mutations have been shown to cause PC/LPC overload and TAG shortage [65, 76], which can lead to Ca^2+^ dyshomeostasis [64], ER stress [77, 78], and abnormal lipid droplet (LD) formation [79–92]. Moreover, the catalytic domain of PLPLA6 was found to have a high intrinsic affinity for LDs and can cause LD clustering independent of its catalytic activity [65]. Interestingly**,** the catalytic domain of PNPLA7, another ER-localized member of the PNPLA family, is also associated with LDs [93, 94], and PNPLA6 may cooperate with PNPLA7 to regulate PC/LPC hydrolyzation and LD homeostasis [62, 63, 94–99]. In brief, PNPLA6 dysfunction leads to abnormal PC/LPC content in the ER membrane, which will cause lipid bilayer stress [100], thereby changing the protein structure and function in the membrane [101], and subsequently triggering ER stress and LD formation [79–92]. If any of these mechanisms fail or ER stress is prolonged, the ER stress response will shift from an adaptive to a pro-apoptotic mechanism [81, 102], which will eventually cause cell death and depletion. This may explain why PNPLA6 deficiency can cause a spectrum of disorders. It is noteworthy that PC has a heterogeneous nature and plays pleiotropic roles, and its overload can produce many harmful effects [103]. Therefore, it cannot be excluded that other mechanisms may contribute to PLPLA6-associated ataxic disorders. Therefore, further studies are required.

### α/β-Hydrolase Domain Containing 12 (ABHD12)

ABHD12 is a metabolic serine hydrolase in the ER membrane [104, 105], which has two main substrates, namely 2-arachidonoyl glycerol (2-AG) [106] and lysophosphatidylserine (LPS) [107]. Importantly, its mutations are linked to neurodegenerative disorders, such as polyneuropathy, hearing loss, ataxia, retinitis pigmentosa, and cataract (PHARC) [16, 108]. Although PHARC syndrome can be clinically variable [109–112], the cerebellum of PHARC subjects is the most atrophied brain region in any case [113].

Mechanistically, studies indicate that ABHD12 deficiency can cause neuroinflammation through very-long-chain LPS [105, 107] or arachidonic acid (AA)-derived lipids [114, 115]. Given that (1) the distribution of ABHD12 in the cerebellum is mainly found in microglia and Purkinje cells, (2) PHARC is characterized by age-dependent microglial activation and demyelination [108], and (3) microglia can cause Purkinje cell degeneration by engulfing and phagocytosing their dendrites [116], It would be very important to reveal how 2-AG and LPS act synergistically in the microglia demyelination process. The endocannabinoid (eCB) system is required for long-term depression (LTD) induction [117–119] and confers neuroprotection against demyelination [120]. However, chronic 2-AG overload can desensitize cannabinoid CB1 receptor signaling, resulting in functional antagonism of the cannabinoid system [121]. Further, LPS can induce inflammation and demyelination through several pathways [122–126]. Interestingly, during inflammation, LDs are required for both generation of AA-derived eicosanoids [127] and efficient phagocytosis by macrophages [128]. Thus, deletion of *ABHD12* may affect LD status in Purkinje and microglia cells, and LD dysregulation can play a key role in the entire pathological process of PHARC. In agreement with this, increased local concentration of DAG (the precursor of 2-AG) at specific ER sites promotes LD formation [129]. Interestingly, DAG also plays a critical role in autophagic flux [130, 131].

### β-Glucosidase 2 (GBA2)

GBA2 is a glucocerebrosidase that mainly hydrolyzes glucosylceramide (GlcCer) to glucose and ceramide [132, 133]. It is ubiquitously expressed [132], but is especially abundant in cerebellar Purkinje cells [134–136]. Subcellularly, GBA2 is localized in the ER and Golgi membranes with its N and C termini facing the cytoplasm [134]. *GBA2* depletion can cause strong or mild locomotor defects in mice [137], while in humans, its mutations are associated with hereditary spastic paraplegia/cerebellar ataxia (SPG46) and Marinesco-Sjögren-like syndrome, both characterized by cerebellar ataxia [138–142].

Regarding the pathogenesis, it has been shown that *GBA2* depletion causes the accumulation of GlcCer outside the lysosomes [132, 143, 144] and leads to aberrant F-actin dynamics [137, 145]. However, the causality and detailed mechanism are unclear. Interestingly, GBA2 has been found to be involved in several neurodegenerative diseases. Its expression was upregulated in Niemann-Pick type C (NP-C) [135, 146] and Gaucher disease [147, 148], and was downregulated in Parkinson’s disease (PD) [149, 150]. The upregulation of GBA2 in NP-C caused defects in sphingolipid targeting from ER to Golgi and lysosomal pH adjustment [146, 151–153], whereas the downregulation of GBA2 in PD was associated with synucleinopathy, which was closely related to autophagic dysfunction [150, 154]. Taken together, GBA2 mutants may cause diseases via a similar mechanism, namely autophagic dysfunction because of GlcCer overload and abnormal lysosomal pH. F-actin dysfunction is probably only a secondary deficiency.

### Elongation of Very Long Chain Fatty Acids-Like 4/5 (ELOVL4/5)

The biosynthesis of very long-chain FAs (VLCFAs) is catalyzed by a four-step reaction cycle; the first and also the rate-limiting step is carried out by the FA elongase family consisting of seven members [155–157], each with substrate specificity [156, 158, 159]. In mammals, while ELOVL5 catalyzes mainly polyunsaturated FAs with 18–20 carbons [156, 160, 161], the substrates of ELOVL4 are both saturated and polyunsaturated FAs with 20–26 carbons [156, 159, 162]. In the cerebellum, ELOVL5 expression is relatively late and is highly concentrated in Purkinje cells [161, 163]. In contrast, the expression of ELOVL4 begins in the embryonic stage, but only at a moderate level in Purkinje cells, and is mainly expressed in oligodendrocytes and other neurons [164].

Mutations in ELOVL4 and ELOVL5 have been linked to spinocerebellar ataxia 34 and 38 (SCA34/38), respectively [161, 165, 166]. SCA34 is a cerebellar ataxia combined with multisystem degeneration [165, 167, 168], while SCA38 is a relatively pure form [161, 169]. Thus far, all the known pathogenic mutations of ELOVL4 and ELOVL5 affect their catalytic sites [161, 170, 171]. However, in the serum of SCA34- and SCA38-affected individuals, only ELOVL5 products were reduced [161, 166]. This observation may reflect different degrees of damage by distinct mutants. ELOVL4 and ELOVL5 are diversely distributed; therefore, they may exert different functions in distinct cell populations. For example, the FA components of myelin lipids in oligodendrocytes are predominantly products of ELOVL4 [170, 172, 173], implicating ELOVL4 is mainly involved in myelination. Notably, dyshomeostasis of VLCFAs is directly related to cerebellar ataxia [174]. Furthermore, alterations in VLCFA-containing lipid species induces a drastic reduction of LDs [175], which also causes ataxia [176, 177]. Overall, in addition to the dominant negative effect of the ELOVL4 mutant on SCA34 [178, 179], abnormal VLCFAs may be the main contributor to SCA34 and SCA38 through various mechanisms, in which LD dyshomeostasis may play a key role.

### Run Domain- and Cysteine-Rich Domain-Containing Beclin-1-Interacting Protein (RUBCN)

Rubicon (RUBCN) is a ubiquitously expressed Beclin1-binding partner that is involved in PI(3)P production. It has been found on late endosomes/lysosomes (LELs) [180–182] and on LC3-associated phagosomes (LAPosome) [183]. It harbors several functional domains and forms different complexes [184, 185]. Particularly, its action in different complexes is functionally and genetically separable [186]. On LELs, Rubicon interacts with Beclin1-VPS34 and inhibits its lipid kinase activity, thus decreasing PI(3)P production for autophagy suppression [187]. On LAPosomes, Rubicon interacts with Beclin1-VPS34 and promotes local production of PI(3)P to recruit downstream conjugation systems for immunosuppression [188, 189].

An ancient mutation that causes the deletion of Rubicon FYVE-like domain is linked to autosomal recessive spinocerebellar ataxia (SCAR15) [190, 191]. Mechanistically, it was thought that mislocalization of Rubicon underlies the pathogenesis [192]. However, how the mislocalization causes SCAR15 is unclear. Rubicon depletion even improves autophagy flux and extends lifespan [154, 193]. It has been reported that proteins containing the FYVE domain can bind to PI(3)P [194, 195]. Therefore, we speculate that through PI(3)P binding, Rubicon FYVE-like domain may play a critical role in positioning Rubicon on both LAPosomes and LELs. Moreover, the Rubicon FYVE-like domain is located within the Rubicon homologous region, which interacts with Rab7 [184, 196], whereas Rab7 plays a key role in the conversion of endosomes to autophagolysosomes [197–199] and cholesterol incorporation into LDs [200]. Thus, depletion of the FYVE-like domain may not only cause LAPosome deficiency and inflammation, but also disrupt downstream fusion between LAPosomes and lysosomes and LD formation due to failure of Rab7 coordination. These factors may contribute to SCAR15 pathogenesis.

### Fatty Acid 2-Hydroxylase (FA2H)

FA2H is a Ceramide 2-hydroxylase located in the ER membrane [201–204]. It catalyzes hydroxylation at the α C position of the N-acyl chain of sphingolipids [202, 205]. In the nervous system, α-hydroxylated sphingolipids are the most abundant lipids in the myelin sheath [203], and the FA components of sphingolipids are predominantly products of ELOVL4 [170, 172, 173, 206], indicating that both FA2H and ELOVL4 play vital roles in myelination.

FA2H mutations are linked to a complicated form of hereditary spastic paraplegia (SPG35) [207, 208], which is often associated with cerebellar ataxia [209–211]. Interestingly, homozygous mutations of ELOVL4 are also found in patients with spastic paraplegia [165]. It has been shown that in the cerebellum, FA2H products from oligodendrocytes are required for long-term myelin sheath maintenance [212], and their absence causes axonal degeneration [213, 214]. In addition, FA2H can form a complex with proteins that are involved in the biosynthesis and metabolism of cholesterol and AA-derived lipids [215–223]. Thus, we speculate that FA2H deficiency can also cause dyshomeostasis of these lipids. In support of this, loss of the *C. elegans* homologue of FA2H inhibits LD formation [224].

### Steroid 5-Alpha-Reductase 3 (SRD5A3)

SRD5A3 is a polyprenol reductase that is necessary for dolichol biosynthesis [225]. Dolichol is the lipid used to build the lipid-linked oligosaccharide precursor, which plays a key role in glycosylation and glycosylphosphatidylinositol (GPI) anchor synthesis [226]. Mutations in SRD5A3 are associated with a new type of CDG characterized by cerebellar ataxia combined with other defects [225, 227, 228].

SRD5A3 is highly expressed in the fetal brain, especially in the cerebellum [227]. *SRD5A3* depletion from the cerebellum causes abnormal granule cell development and motor coordination defects in mice, but only mild N-glycosylation impairment [229]. However, the protein abundance or N-glycosylation level of a subset of glycoproteins with high N-glycans multiplicity per protein decreased [229]. Of note, several proteins described in this review, namely PNPLA6 [61], ELOVL4/5 [171], ABHD12 [105], TMEM30A [230], and NPC1 [231], are N-glycosylated proteins. Thus, impaired glycosylation of these proteins may contribute to *SRD5A3* mutation-induced CDG. Additionally, mutations in several genes involved in GPI anchor synthesis showed reduced myelination and defective Purkinje cell development with progressive ataxia [232–236]. Taken together, these observations demonstrate that N glycosylation disruption and GPI anchor synthesis deficiency can cause developmental impairment of cerebellar granule or Purkinje cells, thereby leading to cerebellar ataxia. Thus, clarification of these signaling pathways may help in the design of new therapeutic strategies for related disorders. For example, glycosylation inhibition can reduce cholesterol accumulation in *NPC1* knockout cells [237].

## Genes Involved in Lipid Scrambling or Flip/Flop

Flippase/floppase and scramblase are involved in the heterogeneous distribution of lipids [238–240]. In this section, we review two of these genes that are involved in lipid flip-flopping and are closely related to cerebellar ataxia.

### Transmembrane Protein 16 k (TMEM16K)

The TMEM16 family consists of ten integral membrane proteins that have diverse functions and are implicated in several human diseases [241–245]. TMEM16K is a Ca^2+^-regulated phospholipid scramblase mainly located in the ER [246, 247]. It is highly expressed in the cerebellum and cerebral cortex [248]. Mutations in TMEM16K are linked to autosomal recessive cerebellar ataxia type 3 (ARCA3) [249–252].

Phospholipids are synthesized and deposited asymmetrically on the cytoplasmic surface of the ER [253, 254]. While the flippase/floppase establishes and maintains the asymmetric membrane structure, the scramblase disrupts the asymmetric status [240, 255–257]. It has been shown that TMEM16K and three G‐protein‐coupled receptors (GPCRs) mediate phospholipid scrambling in the ER membrane [246, 247, 258, 259], and these scramblases are able to scramble all common phospholipids [260, 261]. Interestingly, lipid disequilibrium in the ER membrane is closely related to ER stress [77, 78] and Ca^2+^ homeostasis [262, 263]. Consistent with this, all three GPCR scramblases can cause ER stress and apoptosis when they are dysfunctional [264–266], suggesting that TMEM16K mutants probably cause ARCA3 through the same mechanism. Indeed, TMEM16K defects result in deranged Ca^2+^ signaling [267] and Purkinje cell dysfunction [248, 251, 268]. It is notable that phospholipid disequilibrium of the ER membrane not only causes deranged Ca^2+^ signaling [267], but also LD dyshomeostasis [269, 270], both of which may contribute to ER stress and ultimately lead to cell death [271].

### Transmembrane Protein 30A (TMEM30A)

TMEM30A is an accessory subunit of the heteromeric P4-ATPase complex [272–274]. The human genome encodes 14 members of P4-ATPases, which catalyze the translocation of aminophospholipids across cell membranes to establish phospholipid asymmetry [275, 276]. Some P4-ATPases translocate PS and PE, whereas others are selective for PC [276–279]. Interestingly, the substrate preference of different P4-ATPase members is mainly determined by **t**he accessory subunit, whereas the translocon is formed by transmembrane domains of P4-ATPases [276].

Among the three known accessory subunits, TMEM30A is the most widely expressed and forms a heteromeric complex with 11 of the 14 mammalian P4-ATPases [273, 274]. In particular, TMEM30A can form a complex with ATP8A2 and transports PS and PE across the membrane [230, 280, 281]. Deletion of TMEM30A from mouse cerebellar Purkinje cells was shown to cause early onset cerebellar ataxia [282], while ATP8A2 mutations are associated with CAMRQ syndrome characterized by cerebellar ataxia and quadrupedal locomotion [283–285]. Interestingly, although ATP8A2 is most abundant in the cerebellum, it is not expressed in Purkinje cells, but is expressed in deep cerebellar nuclei [282]. Thus, although both ATP8A2 and TMEM30A deficiencies cause cerebellar ataxia, the underlying cellular mechanisms differ. However, at the molecular level, both mutants cause phospholipid dyshomeostasis, especially loss of PS asymmetry on the plasma membrane, which can cause cell death [286, 287]. Additionally, since TMEM30A is involved in ER exit and proper targeting of several P4-ATPases [230, 282, 288], its dysfunction can cause accumulation of related P4-ATPases in the ER, thereby leading to ER stress. Indeed, in the absence of TMEM30A, the expression levels of CHOP and BiP are elevated in Purkinje cells prior to visible cell loss [282]. Taken together, both lipid dyshomeostasis and proteotoxicity may contribute to the pathology of the disease.

## Genes Involved in Lipid Trafficking

In eukaryotic cells, the bounding membrane of each organelle possesses a characteristic lipid composition, which is required for its identity and function. Therefore, lipids that are synthesized at the ER or taken up from outside of the cell must be transported to various subcellular membranes or locations for their unique functions [289–291]. Thus far, there are two known routes for lipid transfer, namely vesicular and non-vesicular pathways [292–295]. Here, we highlight the genes that are involved in lipid trafficking and are related to cerebellar ataxia.

### Sorting Nexin 14 (SNX14)

Sorting nexins are a large group of proteins, all of which contain a phosphoinositide-binding PX domain [296–298]. Based on its molecular structure, SNX14 is divided into the RGS-PX subfamily [299]. It is an ER transmembrane protein highly expressed in the cerebellum [300, 301]. In the Hungarian Vizsla dog breed, a splice donor site mutation of SNX14 is linked to progressive cerebellar ataxia [302]. In humans, its mutations cause pediatric-onset autosomal-recessive cerebellar ataxia and intellectual disability syndrome, namely SCAR20 [252, 303–305].

SNX14 is reportedly required for the correct flux and storage of neutral lipids among the ER, lysosomes, and LDs [301, 306], and autophagic dysfunction is thought to be involved in the pathogenesis of SCAR20 [307–309]. However, although the yeast homolog of SNX14 was able to link ER to both vacuole and LDs [310, 311], SNX14 only connects ER with LD [301, 306]. At the ER-LD junction, it controls lipid flux from the ER into LD [301, 306, 310–317]. However, it is unknown how autophagy is affected by SNX14.

Failure to package excess lipids into LDs is known to cause lipotoxicity [318, 319]. This mechanism most probably underlies SCAR20. Although the brain displays low levels of LDs under resting conditions [320], there are still many neuronal diseases that are related to LD dysfunction [321–325]. In particular, the neutralization of GRAF1, a GTPase-activating protein that is enriched in Purkinje cells at LD junctions and promotes LD clustering and growth, is associated with subacute cerebellar ataxia [192, 193]. Thus, LD dyshomeostasis may play a critical role in the pathogenesis of SCAR20. As for the autophagic dysfunction under SNX14 deficiency, this may be caused by coupling disorders between LDs and lysosomes [326, 327].

### Niemann-Pick Type C Protein 1 (NPC1)

NPC1 is a large polytopic transmembrane protein of LELs, which is critical for cholesterol trafficking from LELs to the ER, Golgi, and plasma membrane [328–331]. Mutations in the *NPC1* gene can cause cholesterol and glycosphingolipid overload in LELs [146, 332], which can eventually result in NP-C characterized by cerebellar Purkinje cell degeneration [333, 334] and cerebellar ataxia [335, 336]. Although in NP-C, microglia, oligodendrocytes, and GABAergic interneurons are all involved and contribute to Purkinje cell degeneration [337–343], it is believed that autonomous factors cause the susceptibility of Purkinje cells to NPC1 deficiency [338]. However, thus far, the mechanism of this selective vulnerability is unclear.

In the brain, the blood–brain barrier restricts cholesterol in plasma from entering the CNS, therefore, cholesterol must be synthesized locally to meet the demand [344–346]. During development, oligodendrocytes synthesize large quantities of cholesterol for myelination; in adults, glial cells (mostly astrocytes) account for the steady-state production of cholesterol [347], with many proteins involved in the transportation from glia to neurons [331, 348–358]. Inside the cell, both vesicular and non-vesicular pathways operate in parallel to deliver cholesterol from LELs to ER [331, 359, 360]. Among them, NPC1 forms two bridges at the membrane contact sites (MCSs) between LEL and ER to regulate cholesterol egress [360–362], implying that NPC1 deficiency may lead to a defect in cholesterol delivery at MCSs, which can lead to its accumulation in LELs and simultaneous paucity in the ER.

The unavailability of cholesterol in the ER leads to its impaired esterification [358] and hydroxylation [363–366], which in turn will affect its incorporation into LDs [200] and its turnover [367]. In particular, the cytochrome P450 hydroxylases of both *NPC1* deleted mice and NP-C patients become defective in a cerebellum-specific manner [368–370]. Intriguingly, in SCA2 and SCA3, CYP46A1 also becomes defective, and delivery of *CYP46A1* to the cerebellum can prevent Purkinje cell loss and cerebellar atrophy [371]. Coincidently, both *CYP46A1* delivery to SCA3 mice and 14,15-EET (a cytochrome P450 metabolite) treatment of NP-C mice strongly improves autophagic flux [369, 371, 372]. Taken together, these findings suggest that CYP46A1 defects and resultant metabolite dyshomeostasis contribute to NP-C disorder, possibly through autophagic dysfunction, and they can serve as promising therapeutic targets for cerebellar ataxia. Notably, it has been reported that Ca^2+^, eCB, and other signaling pathways also contribute to NP-C pathogenesis [373–375]. These may be downstream or side effects of cytochrome P450 deficiency, which require further verification.

### Vacuolar Protein Sorting 13 Homolog D (VPS13D)

VPS13D belongs to the VPS13 protein family, which consists of four members (VPS13A-D) in eukaryotic cells, and a group of ubiquitously distributed and highly conserved proteins with phospholipid binding properties [376–380]. Mutations in each of the four members can cause movement disorders, among which VPS13D deficiency causes either autosomal recessive spinocerebellar ataxia-4 (SCAR4) [381–384], or hereditary spastic paraplegia (HSP) [384–386], both are characterized by cerebellar ataxia. Thus far, the underlying mechanism is poorly understood.

VPS13A and VPS13C reportedly participate in the transfer of phospholipids between the ER and other organelles in a non-vesicular manner [387]. Interestingly, although VPS13A, VPS13C, and VPS13D show high similarity [376], VPS13D does not have the predicted FFAT motif [388], which is responsible for the binding of VPS13A and VPS13C to VAP on the ER membrane [389], implying that VPS13D is not located at the MCSs between the ER and other organelles. Recently, VPS13D has been shown to be necessary for both mitochondrial dynamics and clearance. Surprisingly, VPS13D did not show clear localization with mitochondrial markers, but co-localized with the lysosomal marker LAMP1 [390], while reduced phosphorylation at S2429 disrupted autophagy flux [391]. Crucially, VPS13D, with decreased phosphorylation at S2080/S2435 and increased phosphorylation at S15, targeted LDs [392]. Overall, it appears that the localization and subcellular rearrangement of VPS13D to lysosomes and LD membranes are involved in the phospholipid exchange between lysosomes and LDs, which may be tightly related to mitochondrial dynamics and clearance [393, 394]. Consistent with this, it was newly reported that loss of VPS13D in Drosophila larval motoneurons does not prevent mitophagy initiation, but causes the accumulation of mitophagy intermediates in cell bodies [395]. In addition, it has been shown that LD plays an essential role in autophagic flux, and autophagosomes appear to form in and around LDs [326, 327]. Thus, VPS13D deficiency may cause autophagic dysfunction by affecting lysosome and LD status. In agreement with this, a disease-causing mutation (N3521S) in the VAB domain of VPS13D blocks its membrane recruitment via adaptor binding [396].

### SCY1-Like 1 (SCYL1)

SCYL1 is a widely expressed and catalytically inactive protein kinase [397]. In the CNS, it is confined to the perikarya of neurons, most prominently in the cerebellar Purkinje cells [397]. In mice, mutations in SCYL1 cause a recessive form of spinocerebellar neurodegeneration characterized by Purkinje cell loss and cerebellar atrophy [398]. In humans, its dysfunction causes SCAR21 characterized by cerebellar ataxia and atrophy in early childhood [399, 400]. Mechanistically, SCYL1 dysfunction has been shown to cause defects in both the nuclear pore [398, 401] and COPI complexes [402, 403]. Interestingly, mutations in the gene encoding the δ subunit of the COPI complex also caused Purkinje cell degeneration [404].

Interestingly, the COPI complex is involved in the maintenance of lipid homeostasis [405, 406]. Arf1/COPI proteins can directly localize to LDs and change the content of phospholipids in the LD membrane and thereby LD surface tension [407, 408]. Variations in LD surface tension affects not only the formation of ER-LD bridges, but also the recruitment of enzymes for lipid synthesis [407, 408]. Particularly, COPI together with a guanine nucleotide exchange factor recruits Rab18 to LD [409]. However, Rab18 has been shown to play a key role in LD growth and in maintaining ER-LD contact [410]. Taken together, these observations indicate that SCYL1 dysfunction may cause SCAR21 by inducing LD dyshomeostasis.

## Calcium and Lipid Dyshomeostasis

The inositol 1,4,5-triphosphate receptors (ITPRs) are calcium release channels located in the ER membrane [411–413]. In mammals, there are three ITPR subtypes (ITPR1-3) [414, 415]. ITPR1 is the major subtype in the CNS and is predominantly concentrated in the cerebellar Purkinje cells [416–419]. Its mutations can cause Purkinje cell malfunction [420, 421], and are associated with several human disorders characterized by cerebellar ataxia [422–429]. Although, it is well documented that their pathogenesis is closely related to aberrant Ca^2+^ homeostasis [422, 424, 430, 431], the underlying pathogenic mechanism has not been well defined.

Intriguingly, ITPR1 is physically associated with STARD13 [432–435], which was found to be a lipid binding protein located in proximity to LDs overlapping with mitochondria [436]. STARD13 participates in both synthesis and transfer of phospholipids [436, 437], suggesting that Ca^2+^ signaling and lipid homeostasis are closely coupled with each other. In line with this, ITPR1 depletion caused lipid dyshomeostasis in both Drosophila and mice [438, 439]. Additionally, Ca^2+^ downregulation in the ER via calreticulin deletion increases lipid synthesis via the SCAP-SREBP signaling pathway [440], while Ca^2+^ upregulation in the ER via TMCO1 deletion reduces the number of LDs and the TAG content through ER stress-associated degradation of diacylglycerol acyltransferase 2 [441, 442]. Moreover, the ATAXIN2 mutant causing SCA2 binds with ITPR1 and results in deranged Ca^2+^ signaling [32, 443]. In addition, the brain lipidome of SCA2 patients showed prominent abnormalities in ceramide and sphingosine levels, and many enzymes, such as ELOVL4, serine palmitoyltransferase long-chain base subunit 2, and ceramide synthase 2, were affected.

Recently Rodríguez-Pascau et al. found that frataxin (a small mitochondrial protein encoded by nuclear genome) is present in ER-mitochondria associated membranes (MAMs) where it interacts with ITPR1 (IP3R) and GRP75, and that frataxin deficiency causes an impairment in both the ER-mitochondria communication and in the dysregulation of Ca^2+^ homeostasis [444]. Of particular importance, frataxin deficiency leads to Friedreich ataxia (FRDA), the most common hereditary ataxia in humans [2, 445]. Considering that frataxin directly interacts with ITPR1 and that accumulation of LDs and increased lipogenesis have been previously described in fibroblasts of FRDA patients, cardiomyocytes of mice and glial cells of Drosophila [446–448], it is conceivable that ITPR1-mediated signals may contribute to frataxin deficiency-triggered lipid dyshomeostasis together with other mechanisms [449, 450], which probably also occur in Purkinje and other cerebellar cells. In line with this notion, frataxin (like ITPR1) is highly expressed in cerebellar Purkinje neuron and large principal neurons of dentate nuclei (DN) [451]. Moreover, the neurological symptoms of Friedreich ataxia have been shown to be a consequence of lesions in the dentate nuclei (DN) and Purkinje cells of the cerebellum as well as degeneration of the large sensory neurons of the dorsal root ganglia and of the spinocerebellar tracts [445, 452]. Collectively, it seems that ITPR1 may affect the lipid landscape through several different pathways. Conversely, the ITPR1 function is also regulated by surrounding lipids [262, 453–461]. Overall, Ca^2+^ and lipid dysregulation may act in a synergistic manner to contribute to ataxic pathogenesis and result in neuronal cell death and cerebellar atrophy.

**Unfolded Protein Response (UPR)**/**Endoplasmic Reticulum-Associated Protein Degradation** (**ERAD) and Lipid Dyshomeostasis.**

Studies have shown that both UPR and ERAD are essential for maintaining lipid homeostasis [77, 462, 463]. While lipid bilayer stress can activate all three branches of UPR transducers (IRE1α, PERK, and ATF6) to buffer lipid imbalance [78, 464–467], ERAD is responsible for degrading many enzymes involved in lipid synthesis, degradation, and secretion [42]. In particular, ERAD is responsible for the degradation of 3-hydroxy-3-methylglutaryl-coenzyme A reductase (HMGCR), adipose triglyceride lipase (ATGL) [468–472], and proteins from ER to LD [473]. All these are crucial for maintaining LD homeostasis. Additionally, ERAD is responsible for COX2 degradation, which plays a critical role in eCB hydrolysis and PGE2 production [474, 475], and for ITPR1 degradation [476–481], which is involved in lipid metabolism through Ca^2+^ mobilization [438, 439].

Furthermore, several components of the ERAD or UPR machinery have been linked to cerebellar ataxia**.** SEL1L deficiency leads to a canine progressive early-onset cerebellar ataxia [482, 483]. Loss-of-function mutations in DNAJC3 and BAP cause multisystemic neurodegeneration and Marinesco-Sjögren syndrome, respectively [484–487]. SEL1L, which forms the ERAD complex with HRD1 and is involved in the degradation of LDLR and HMGCR [468–470, 488], forms another complex with lipoprotein lipase (LPL) and lipase maturation factor 1, which is essential for the secretion of LPL [482]. DNAJC3 and BAP form a complex with HSPA5 [489–491], which plays a key role in lipid metabolism [492–495] by interacting with the ER stress transducers and SREBP-SCAP complex [494–497]. In particular, DNAJC3 can directly bind and inhibit PERK [491, 498]. Interestingly, PERK harbors intrinsic lipid kinase activity, which favors the conversion of DAG to PA [499]. Furthermore, PERK inhibition can reduce stearoyl-CoA desaturase 1 and fatty acid synthase expression [500]. Overall, these observations clearly indicate that URP and ERAD are both promising therapeutic targets for cerebellar ataxia.

## Conclusion

**Fig. 2:**
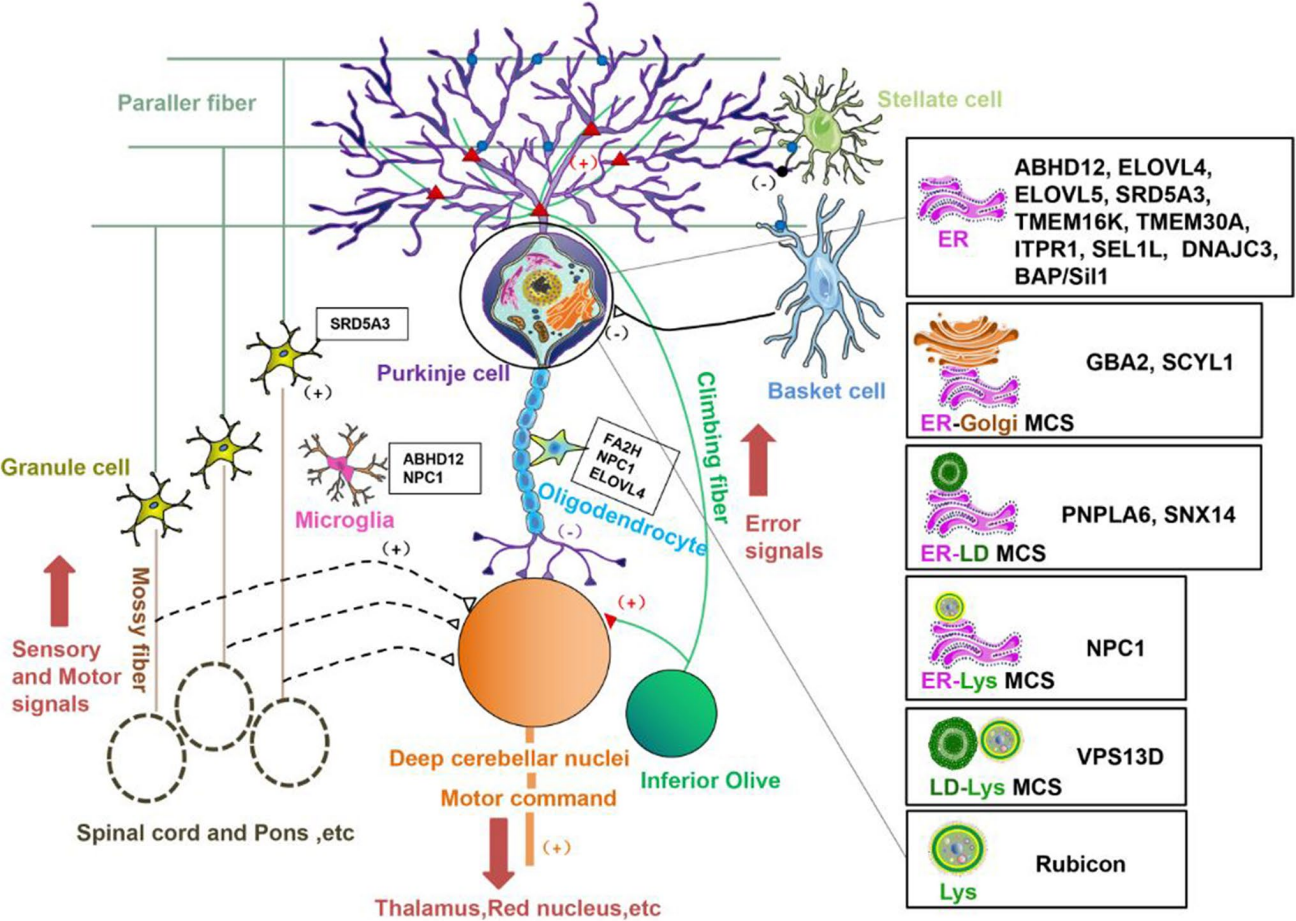
Schematic illustration of the molecules involved in lipid homeostasis and cerebellar ataxia and their localization in Purkinje cells. In total, 18 molecules involved in cerebellar lipid homeostasis are illustrated. Except FA2H, all molecules are expressed in Purkinje cells. In addition, NPC1 in microglia and oligodendrocytes, ELOVL4 in oligodendrocytes, ABHD12 in microglia, and SRD5A3 in granule cells, are also abundant. In Purkinje cells, molecular localization is shown, indicating that most are located in the ER membrane or at MCSs between ER and other organelles

**Fig. 3:**
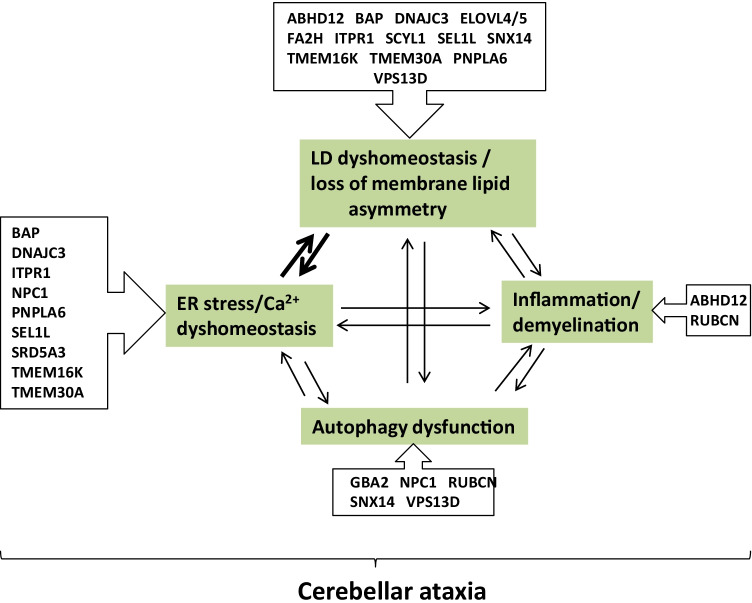
Possible pathological mechanisms of cerebellar ataxia caused by gene defects related to lipid imbalance. These gene defects mainly lead to four closely related pathological processes, namely LD dyshomeostasis, ER stress, Autophagy dysfunction and Inflammation. In particular, most of them cause LD dyshomeostasis and ER stress, suggesting ER-LD system is a key target for the treatment of cerebellar ataxia

We hypothesize that cerebellar Purkinje cells are especially susceptible to abnormal turnover of a subset of lipids. Most related genes are highly expressed in Purkinje cells, which may explain why Purkinje cells are more susceptible to lipid dyshomeostasis caused by their defects. Although some genes are ubiquitously expressed, it has been suggested that distinct cells may have different compensatory functions for their defects [485]. This may be the case for Purkinje cells due to their complicated dendritic trees and long myelinated axons. These lipids mainly include PC/LPC, PS/LPS, PI(3)P, cholesterol, sphingolipids, VLCFAs, cannabinoids, and dolichol, and more than a dozen genes are involved in or closely related to their synthesis, degradation, storage, and distribution (Table 1). Crucially, the dysfunction of these genes or the lipid imbalances they induce are closely related to ER stress, autophagy, or inflammation/demyelination, which may eventually lead to cell death and cerebellar ataxia. Intriguingly, all but two of the proteins encoded by these genes are located in the ER membrane or other organelles, particularly at the MCSs between the ER and lysosome, LD, and Golgi (Fig. 2). The other two proteins are located in the ER lumen, but are also closely related to the regulation of ER membrane proteins in lipid metabolism. Lipid synthesis, deposition, distribution, and degradation are generally in the same streamline. In particular, many of these proteins are involved in or closely related to the maintenance of LD homeostasis, indicating that LD dyshomeostasis plays a key role in the pathogenesis of ataxic disorders. LDs are derived from the ER and encounter the ER and many other organelles. Accumulating evidence shows that LDs are not limited to the inert storage of excess lipids, but also dynamically participate in many cellular functions. Therefore, in addition to their roles in ER stress, autophagy, and inflammatory processes, LDs may play important roles in Purkinje cells (Fig. 3), which requires further investigation. Overall, our review implies that the ER-LD system is the core facility for maintaining lipid homeostasis in cerebellar Purkinje cells, and its defects, caused by dysregulation of many lipid species, can lead to cerebellar ataxia. We believe that these findings will provide a valuable reference for future diagnosis and treatment of ataxic disorders.

**Table 1:** Proteins implicated in lipid homeostasis and cerebellar ataxia. Overview of the proteins, their targets, and the related disorders. Eighteen proteins were divided into five groups according to their functions. All proteins are involved in maintaining lipid homeostasis

Classification	Protein	Target molecules	Disease	Cell type	References
Lipid Lipogenesis or lipolysis	PNPLA6	PC; LPC	Cerebellar Ataxia	Purkinje cell	Wiethoff et al., 2017
	ABHD12	2-AG; LPS	PHARC	Purkinje cell and microglia	Fiskerstrand et al., 2010; Blankman et al., 2013
	GBA2	GlcCer; GalCer	SPG46; MSS	Purkinje cell	Martin et al., 2013; Hammer et al., 2013
	ELOVL4	VLC-SFA; VLC-PUFA	SCA34	Purkinje cell & other neurons	Cadieux-Dion et al., 2014; Ozaki et al., 2015
	ELOVL5	VLC-PUFA	SCA38	Purkinje cells & other neurons	Di Gregorio et al., 2014
	Rubicon	PI(3)P	SCAR15	Ubiquitous	Assoum et al., 2010; Seidahmed et al., 2020
	FA2H	α-hydroxylated FAs	SPG35	Oligodendrocyte	Edvardson et al., 2008; Rattay et al., 2019
	SRD5A3	dolichol	CDG	Granule cell & Purkinje cell	Cantagrel et al., 2010; Kasapkara et al., 2012
lipid scrambling or flip/flop	TMEM16K	Phospholipids	SCAR10 (ARCA3, ATX-ANO10)	Purkinje cell	Vermeer et al., 2010; Nanetti et al., 2019
	TMEM30A	PS; PE	Cerebellar ataxia	Purkinje cell	Yang et al., 2018
Lipid trafficking	SNX14	ACSL3;TAG; phospholipids	SCAR20 (ATX-SNX14)	Ubiquitous	Thomas et al., 2014; Shukla et al., 2017
	NPC1	Cholesterol; Sphingolipids	NP-C	Purkinje cell Oligodendrocyte	Vanier, 2010
	VPS13D	PS; PE	SCAR4; HSP	Microglia Ubiquitous	Seong et al., 2018; Koh et al., 2020
	SCYL1	Phospholipids	SCAR21	Purkinje cells & other neurons	Schmidt et al., 2015; Shohet et al., 2019
Calcium and lipid	ITPR1	PG, TAG	SCA15/16; SCA29	Purkinje cell	Gerber et al., 2016; Van de Leemput et al., 2007; Zambonin et al., 2017; van Dijk et al., 2017
UPR/ERAD and lipid	SEL1L	LDLR; HMGCR; LPL	Cerebellar ataxia	Ubiquitous	Kyöstilä et al., 2012; Sha et al., 2014;
	DNAJC3& BAP/Sil1	DAG; SCD1; FASN	Cerebellar ataxia; MSS	Ubiquitous	Kizhakkedath et al., 2018; Bobrovnikova-Marjon et al., 2012; Bobrovnikova-Marjon et al., 2008

*PNPLA6* patatin-like phospholipase domain-containing protein 6; *ABHD12* α/β-hydrolase domain containing 12; *GBA2* β-glucosidase 2; *ELOVL4* elongation of very long chain fatty acids-like 4; *ELOVL5* elongation of very long chain fatty acids-like 5; *Rubicon (RUBCN)* run domain- and cysteine-rich domain-containing beclin-1-interacting protein; *FA2H* fatty acid 2-hydroxylase; *SRD5A3* steroid 5-alpha-reductase 3; *TMEM16K* transmembrane protein 16 K; *TMEM30A* transmembrane protein 30A; *SNX14* sorting nexin 14; *NPC1* Niemann-Pick type C protein 1; *VPS13D* vacuolar protein sorting 13 homolog D; *SCYL1* SCY1-like 1; *ITPR1* inositol 1,4,5-triphosphate receptor subtype 1; *SEL1L* suppressor of lin12-like; *DNAJC3* DNAJ/HSP40 homolog, subfamily c, member 3; *BAP* BIP-associated protein; *PC* phosphatidylcholine; *LPC* lysophosphatidylcholine; *2-AG* 2-arachidonoyl glycerol; *PS* phosphatidylserine; *LPS* lysophosphatidylserine; *PE* phosphatidylethanolamine; *PG* phosphatidylglycerol; *GlcCer* glucosylceramide; *GalCer* galactosylceramide; *VLC-SFA* very long chain saturated fatty acid; *VLC-PUFA* very long chain polyunsaturated fatty acid; *PI(3)P* phosphatidylinositol-3-phosphate; *TAG* triacylglycerol; *DAG* diacylglycerol; *ACSL3* long chain fatty acyl-CoA ligase 3; *LDLR* low-density lipoproteins receptor; *HMGCR* 3-hydroxy-3-methylglutaryl-coenzyme A reductase; *LPL* lipoprotein lipase; *SCD1* stearoyl-CoA desaturase 1; *FASN* fatty acid synthase; *PHARC* polyneuropathy, hearing loss, ataxia, retinitis pigmentosa, and cataract; *SPG46* autosomal recessive spastic paraplegia-46; *MSS* Marinesco-Sjogren syndrome; *SCA34* spinocerebellar ataxia-34; *SCA38* spinocerebellar ataxia-38; *SCAR15* autosomal recessive spinocerebellar ataxia-15; *SPG35* autosomal recessive spastic paraplegia-35; *CDG* congenital disorder of glycosylation; *SCAR10* autosomal recessive spinocerebellar ataxia-10; *SCAR20* autosomal recessive spinocerebellar ataxia-20; *NP-C* Niemann-Pick disease type C; *SCAR4* autosomal recessive spinocerebellar ataxia-4; *HSP* hereditary spastic paraplegia; *SCAR21* autosomal recessive spinocerebellar ataxia-21; *SCA15/16* spinocerebellar ataxia-15/16; *SCA29* spinocerebellar ataxia-29

## Supplementary Information

Below is the link to the electronic supplementary material.

Supplementary file1 (DOCX 71 KB)

## Data Availability

Not applicable.

## References

[1] Schmahmann JD (2004). Disorders of the cerebellum: ataxia, dysmetria of thought, and the cerebellar cognitive affective syndrome. J Neuropsychiatry Clin Neurosci.

[2] Taroni F, Didonato S (2004). Pathways to motor incoordination: the inherited ataxias. Nat Rev Neurosci.

[3] Lim J, Hao T, Shaw C, Patel AJ, Szabo G, Rual JF, Fisk CJ, Li N (2006). A protein-protein interaction network for human inherited ataxias and disorders of Purkinje cell degeneration. Cell.

[4] Pandolfo M, Manto M (2013). Cerebellar and afferent ataxias. Continuum (Minneap Minn).

[5] Klockgether T (2018). Sporadic adult-onset ataxia. Handb Clin Neurol.

[6] Lieto M, Roca A, Santorelli FM, Fico T, De Michele G, Bellofatto M, Sacca F, De Michele G (2019). Degenerative and acquired sporadic adult onset ataxia. Neurol Sci.

[7] Albin RL (2003). Dominant ataxias and Friedreich ataxia: an update. Curr Opin Neurol.

[8] Manto M, Marmolino D (2009). Cerebellar ataxias. Curr Opin Neurol.

[9] Klockgether T (2011). Update on degenerative ataxias. Curr Opin Neurol.

[10] Manto M, Gandini J, Feil K, Strupp M (2020). Cerebellar ataxias: an update. Curr Opin Neurol.

[11] Zanni G, Bertini E (2018). X-linked ataxias. Handb Clin Neurol.

[12] Coarelli G, Brice A, Durr A (2018) Recent advances in understanding dominant spinocerebellar ataxias from clinical and genetic points of view. F1000Res 7. 10.12688/f1000research.15788.1 10.12688/f1000research.15788.1 PMC6234732 30473770

[13] Gennarino VA, Palmer EE, Mcdonell LM, Wang L, Adamski CJ, Koire A, See L, Chen CA (2018). A Mild PUM1 Mutation Is Associated with Adult-Onset Ataxia, whereas Haploinsufficiency Causes Developmental Delay and Seizures. Cell.

[14] Buijsen R, Toonen L, Gardiner SL, van Roon-Mom W (2019). Genetics, Mechanisms, and Therapeutic Progress in Polyglutamine Spinocerebellar Ataxias. Neurotherapeutics.

[15] Beaudin M, Matilla-Duenas A, Soong BW, Pedroso JL, Barsottini OG, Mitoma H, Tsuji S, Schmahmann JD (2019). The Classification of Autosomal Recessive Cerebellar Ataxias: a Consensus Statement from the Society for Research on the Cerebellum and Ataxias Task Force. Cerebellum.

[16] Ruano L, Melo C, Silva MC, Coutinho P (2014). The global epidemiology of hereditary ataxia and spastic paraplegia: a systematic review of prevalence studies. Neuroepidemiology.

[17] Coutinho P, Ruano L, Loureiro JL, Cruz VT, Barros J, Tuna A, Barbot C, Guimaraes J (2013). Hereditary ataxia and spastic paraplegia in Portugal: a population-based prevalence study. Jama Neurol.

[18] Synofzik M, Nemeth AH (2018). Recessive ataxias. Handb Clin Neurol.

[19] Soong BW, Paulson HL (2007). Spinocerebellar ataxias: an update. Curr Opin Neurol.

[20] Mundwiler A, Shakkottai VG (2018). Autosomal-dominant cerebellar ataxias. Handb Clin Neurol.

[21] Palau F, Espinos C (2006). Autosomal recessive cerebellar ataxias. Orphanet J Rare Dis.

[22] Beaudin M, Klein CJ, Rouleau GA, Dupre N (2017). Systematic review of autosomal recessive ataxias and proposal for a classification. Cerebellum Ataxias.

[23] Al-Muhaizea MA, Almutairi F, Almass R, Alharthi S, Aldosary MS, Alsagob M, Alodaib A, Colak D (2018). A Novel Homozygous Mutation in SPTBN2 Leads to Spinocerebellar Ataxia in a Consanguineous Family: Report of a New Infantile-Onset Case and Brief Review of the Literature. Cerebellum.

[24] Marquer A, Barbieri G, Perennou D (2014). The assessment and treatment of postural disorders in cerebellar ataxia: a systematic review. Ann Phys Rehabil Med.

[25] Klockgether T, Mariotti C, Paulson HL (2019). Spinocerebellar ataxia. Nat Rev Dis Primers.

[26] Ashizawa T, Oz G, Paulson HL (2018). Spinocerebellar ataxias: prospects and challenges for therapy development. Nat Rev Neurol.

[27] Friedrich J, Kordasiewicz HB, O'Callaghan B, Handler HP, Wagener C, Duvick L, Swayze EE, Rainwater O et al (2018) Antisense oligonucleotide-mediated ataxin-1 reduction prolongs survival in SCA1 mice and reveals disease-associated transcriptome profiles. JCI Insight 3(21) 10.1172/jci.insight.123193 10.1172/jci.insight.123193 PMC6238731 30385727

[28] Scoles DR, Meera P, Schneider MD, Paul S, Dansithong W, Figueroa KP, Hung G, Rigo F (2017). Antisense oligonucleotide therapy for spinocerebellar ataxia type 2. Nature.

[29] Mcloughlin HS, Moore LR, Chopra R, Komlo R, Mckenzie M, Blumenstein KG, Zhao H, Kordasiewicz HB (2018). Oligonucleotide therapy mitigates disease in spinocerebellar ataxia type 3 mice. Ann Neurol.

[30] Toonen L, Rigo F, van Attikum H, van Roon-Mom W (2017). Antisense Oligonucleotide-Mediated Removal of the Polyglutamine Repeat in Spinocerebellar Ataxia Type 3 Mice. Mol Ther Nucleic Acids.

[31] Hisatsune C, Hamada K (1865). Mikoshiba K (2018) Ca(2+) signaling and spinocerebellar ataxia. Biochim Biophys Acta Mol Cell Res.

[32] Kasumu AW, Liang X, Egorova P, Vorontsova D, Bezprozvanny I (2012). Chronic suppression of inositol 1,4,5-triphosphate receptor-mediated calcium signaling in cerebellar purkinje cells alleviates pathological phenotype in spinocerebellar ataxia 2 mice. J Neurosci.

[33] Egorova PA, Bezprozvanny IB (2019). Molecular Mechanisms and Therapeutics for Spinocerebellar Ataxia Type 2. Neurotherapeutics.

[34] Mark MD, Schwitalla JC, Groemmke M, Herlitze S (2017). Keeping Our Calcium in Balance to Maintain Our Balance. Biochem Biophys Res Commun.

[35] Synofzik M, Puccio H, Mochel F, Schols L (2019). Autosomal Recessive Cerebellar Ataxias: Paving the Way toward Targeted Molecular Therapies. Neuron.

[36] Bushart DD, Murphy GG, Shakkottai VG (2016). Precision medicine in spinocerebellar ataxias: treatment based on common mechanisms of disease. Ann Transl Med.

[37] Bushart DD, Shakkottai VG (2019). Ion channel dysfunction in cerebellar ataxia. Neurosci Lett.

[38] Bushart DD, Chopra R, Singh V, Murphy GG, Wulff H, Shakkottai VG (2018). Targeting potassium channels to treat cerebellar ataxia. Ann Clin Transl Neurol.

[39] Boy N, Mengler K, Heringer-Seifert J, Hoffmann GF, Garbade SF, Kolker S (2021). Impact of newborn screening and quality of therapy on the neurological outcome in glutaric aciduria type 1: a meta-analysis. Genet Med.

[40] Jacquemyn J, Cascalho A, Goodchild RE (2017). The ins and outs of endoplasmic reticulum-controlled lipid biosynthesis. Embo Rep.

[41] Bell RM, Ballas LM, Coleman RA (1981). Lipid topogenesis. J Lipid Res.

[42] Stevenson J, Huang EY, Olzmann JA (2016). Endoplasmic Reticulum-Associated Degradation and Lipid Homeostasis. Annu Rev Nutr.

[43] Hannun YA, Luberto C, Argraves KM (2001). Enzymes of sphingolipid metabolism: from modular to integrative signaling. Biochemistry-Us.

[44] Futerman AH (2006). Intracellular trafficking of sphingolipids: relationship to biosynthesis. Biochim Biophys Acta.

[45] Parashuraman S, D'Angelo G (2019). Visualizing sphingolipid biosynthesis in cells. Chem Phys Lipids.

[46] van Meer G, de Kroon AI (2011). Lipid map of the mammalian cell. J Cell Sci.

[47] Balla T, Sengupta N (1865). Kim YJ (2020) Lipid synthesis and transport are coupled to regulate membrane lipid dynamics in the endoplasmic reticulum. Biochim Biophys Acta Mol Cell Biol Lipids.

[48] Quiroga AD, Lehner R (2011). Role of endoplasmic reticulum neutral lipid hydrolases. Trends Endocrinol Metab.

[49] Joensuu M, Wallis TP, Saber SH, Meunier FA (2020). Phospholipases in neuronal function: A role in learning and memory?. J Neurochem.

[50] Sharpe HJ, Stevens TJ, Munro S (2010). A comprehensive comparison of transmembrane domains reveals organelle-specific properties. Cell.

[51] van Meer G, Voelker DR, Feigenson GW (2008). Membrane lipids: where they are and how they behave. Nat Rev Mol Cell Biol.

[52] Casares D, Escriba PV, Rossello CA (2019) Membrane Lipid Composition: Effect on Membrane and Organelle Structure, Function and Compartmentalization and Therapeutic Avenues. Int J Mol Sci 20(9). 10.3390/ijms20092167 10.3390/ijms20092167 PMC6540057 31052427

[53] Gibellini F, Smith TK (2010). The Kennedy pathway–De novo synthesis of phosphatidylethanolamine and phosphatidylcholine. IUBMB Life.

[54] Lagace TA (1833). Ridgway ND (2013) The role of phospholipids in the biological activity and structure of the endoplasmic reticulum. Biochim Biophys Acta.

[55] Li Z, Vance DE (2008). Phosphatidylcholine and choline homeostasis. J Lipid Res.

[56] Walkey CJ, Shields DJ, Vance DE (1999). Identification of three novel cDNAs for human phosphatidylethanolamine N-methyltransferase and localization of the human gene on chromosome 17p11.2. Biochim Biophys Acta.

[57] Zhang J, Zhu H, Yang W, Shaw GM, Lammer EJ, Finnell RH (2006). Phosphatidylethanolamine N-methyltransferase (PEMT) gene polymorphisms and risk of spina bifida. Am J Med Genet A.

[58] Zhu X, Mar MH, Song J, Zeisel SH (2004). Deletion of the Pemt gene increases progenitor cell mitosis, DNA and protein methylation and decreases calretinin expression in embryonic day 17 mouse hippocampus. Brain Res Dev Brain Res.

[59] Kienesberger PC, Oberer M, Lass A, Zechner R (2009). Mammalian patatin domain containing proteins: a family with diverse lipolytic activities involved in multiple biological functions. J Lipid Res.

[60] Zaccheo O, Dinsdale D, Meacock PA, Glynn P (2004). Neuropathy target esterase and its yeast homologue degrade phosphatidylcholine to glycerophosphocholine in living cells. J Biol Chem.

[61] Lush MJ, Li Y, Read DJ, Willis AC, Glynn P (1998). Neuropathy target esterase and a homologous Drosophila neurodegeneration-associated mutant protein contain a novel domain conserved from bacteria to man. Biochem J.

[62] van Tienhoven M, Atkins J, Li Y, Glynn P (2002). Human neuropathy target esterase catalyzes hydrolysis of membrane lipids. J Biol Chem.

[63] Quistad GB, Barlow C, Winrow CJ, Sparks SE, Casida JE (2003). Evidence that mouse brain neuropathy target esterase is a lysophospholipase. Proc Natl Acad Sci U S A.

[64] Sunderhaus ER, Law AD, Kretzschmar D (2019). ER responses play a key role in Swiss-Cheese/Neuropathy Target Esterase-associated neurodegeneration. Neurobiol Dis.

[65] Chang P, He L, Wang Y, Heier C, Wu Y, Huang F (2019) Characterization of the Interaction of Neuropathy Target Esterase with the Endoplasmic Reticulum and Lipid Droplets. Biomolecules 9(12). 10.3390/biom9120848 10.3390/biom9120848 PMC6995513 31835418

[66] Moser M, Stempfl T, Li Y, Glynn P, Buttner R, Kretzschmar D (2000). Cloning and expression of the murine sws/NTE gene. Mech Dev.

[67] Akassoglou K, Malester B, Xu J, Tessarollo L, Rosenbluth J, Chao MV (2004). Brain-specific deletion of neuropathy target esterase/swisscheese results in neurodegeneration. Proc Natl Acad Sci U S A.

[68] Topaloglu AK, Lomniczi A, Kretzschmar D, Dissen GA, Kotan LD, Mcardle CA, Koc AF, Hamel BC (2014). Loss-of-function mutations in PNPLA6 encoding neuropathy target esterase underlie pubertal failure and neurological deficits in Gordon Holmes syndrome. J Clin Endocrinol Metab.

[69] Wiethoff S, Bettencourt C, Paudel R, Madon P, Liu YT, Hersheson J, Wadia N, Desai J (2017). Pure Cerebellar Ataxia with Homozygous Mutations in the PNPLA6 Gene. Cerebellum.

[70] Hufnagel RB, Arno G, Hein ND, Hersheson J, Prasad M, Anderson Y, Krueger LA, Gregory LC (2015). Neuropathy target esterase impairments cause Oliver-McFarlane and Laurence-Moon syndromes. J Med Genet.

[71] Deik A, Johannes B, Rucker JC, Sanchez E, Brodie SE, Deegan E, Landy K, Kajiwara Y (2014). Compound heterozygous PNPLA6 mutations cause Boucher-Neuhauser syndrome with late-onset ataxia. J Neurol.

[72] Synofzik M, Gonzalez MA, Lourenco CM, Coutelier M, Haack TB, Rebelo A, Hannequin D, Strom TM (2014). PNPLA6 mutations cause Boucher-Neuhauser and Gordon Holmes syndromes as part of a broad neurodegenerative spectrum. Brain.

[73] Rainier S, Bui M, Mark E, Thomas D, Tokarz D, Ming L, Delaney C, Richardson RJ (2008). Neuropathy target esterase gene mutations cause motor neuron disease. Am J Hum Genet.

[74] Kmoch S, Majewski J, Ramamurthy V, Cao S, Fahiminiya S, Ren H, Macdonald IM, Lopez I (2015). Mutations in PNPLA6 are linked to photoreceptor degeneration and various forms of childhood blindness. Nat Commun.

[75] Li Y, Dinsdale D, Glynn P (2003). Protein domains, catalytic activity, and subcellular distribution of neuropathy target esterase in Mammalian cells. J Biol Chem.

[76] Sunderhaus ER, Law AD, Kretzschmar D (2019). Disease-Associated PNPLA6 Mutations Maintain Partial Functions When Analyzed in Drosophila. Front Neurosci.

[77] Thibault G, Shui G, Kim W, Mcalister GC, Ismail N, Gygi SP, Wenk MR, Ng DT (2012). The membrane stress response buffers lethal effects of lipid disequilibrium by reprogramming the protein homeostasis network. Mol Cell.

[78] Volmer R, van der Ploeg K, Ron D (2013). Membrane lipid saturation activates endoplasmic reticulum unfolded protein response transducers through their transmembrane domains. Proc Natl Acad Sci U S A.

[79] Fei W, Wang H, Fu X, Bielby C, Yang H (2009). Conditions of endoplasmic reticulum stress stimulate lipid droplet formation in Saccharomyces cerevisiae. Biochem J.

[80] Lee JS, Mendez R, Heng HH, Yang ZQ, Zhang K (2012). Pharmacological ER stress promotes hepatic lipogenesis and lipid droplet formation. Am J Transl Res.

[81] Jarc E, Petan T (2019). Lipid Droplets and the Management of Cellular Stress. Yale J Biol Med.

[82] Jacquier N, Choudhary V, Mari M, Toulmay A, Reggiori F, Schneiter R (2011). Lipid droplets are functionally connected to the endoplasmic reticulum in Saccharomyces cerevisiae. J Cell Sci.

[83] Nettebrock NT (1865). Bohnert M (2020) Born this way - Biogenesis of lipid droplets from specialized ER subdomains. Biochim Biophys Acta Mol Cell Biol Lipids.

[84] Fujimoto T, Parton RG (2011) Not just fat: the structure and function of the lipid droplet. Cold Spring Harb Perspect Biol 3(3) 10.1101/cshperspect.a004838 10.1101/cshperspect.a004838 PMC3039932 21421923

[85] Chorlay A, Monticelli L, Verissimo FJ, Ben MK, Ajjaji D, Wang S, Johnson E, Beck R (2019). Membrane Asymmetry Imposes Directionality on Lipid Droplet Emergence from the ER. Dev Cell.

[86] Ridgway ND, Byers DM, Cook HW, Storey MK (1999). Integration of phospholipid and sterol metabolism in mammalian cells. Prog Lipid Res.

[87] Yeagle PL (1989). Lipid regulation of cell membrane structure and function. Faseb J.

[88] Tabas I (2002). Consequences of cellular cholesterol accumulation: basic concepts and physiological implications. J Clin Invest.

[89] Lange Y, Tabei SM, Ye J, Steck TL (2013). Stability and stoichiometry of bilayer phospholipid-cholesterol complexes: relationship to cellular sterol distribution and homeostasis. Biochemistry-Us.

[90] Lagace TA (2015). Phosphatidylcholine: Greasing the Cholesterol Transport Machinery. Lipid Insights.

[91] Choudhary V, Golani G, Joshi AS, Cottier S, Schneiter R, Prinz WA, Kozlov MM (2018). Architecture of Lipid Droplets in Endoplasmic Reticulum Is Determined by Phospholipid Intrinsic Curvature. Curr Biol.

[92] Guo Y, Walther TC, Rao M, Stuurman N, Goshima G, Terayama K, Wong JS, Vale RD (2008). Functional genomic screen reveals genes involved in lipid-droplet formation and utilization. Nature.

[93] Chang P, Sun T, Heier C, Gao H, Xu H, Huang F (2020). Interaction of the Lysophospholipase PNPLA7 with Lipid Droplets through the Catalytic Region. Mol Cells.

[94] Heier C, Kien B, Huang F, Eichmann TO, Xie H, Zechner R, Chang PA (2017). The phospholipase PNPLA7 functions as a lysophosphatidylcholine hydrolase and interacts with lipid droplets through its catalytic domain. J Biol Chem.

[95] Wilson PA, Gardner SD, Lambie NM, Commans SA, Crowther DJ (2006). Characterization of the human patatin-like phospholipase family. J Lipid Res.

[96] Kienesberger PC, Lass A, Preiss-Landl K, Wolinski H, Kohlwein SD, Zimmermann R, Zechner R (2008). Identification of an insulin-regulated lysophospholipase with homology to neuropathy target esterase. J Biol Chem.

[97] Cnop M, Hannaert JC, Hoorens A, Eizirik DL, Pipeleers DG (2001). Inverse relationship between cytotoxicity of free fatty acids in pancreatic islet cells and cellular triglyceride accumulation. Diabetes.

[98] Listenberger LL, Han X, Lewis SE, Cases S, Farese RJ, Ory DS, Schaffer JE (2003). Triglyceride accumulation protects against fatty acid-induced lipotoxicity. Proc Natl Acad Sci U S A.

[99] Thorn K, Bergsten P (2010). Fatty acid-induced oxidation and triglyceride formation is higher in insulin-producing MIN6 cells exposed to oleate compared to palmitate. J Cell Biochem.

[100] Halbleib K, Pesek K, Covino R, Hofbauer HF, Wunnicke D, Hanelt I, Hummer G, Ernst R (2017). Activation of the Unfolded Protein Response by Lipid Bilayer Stress. Mol Cell.

[101] Ernst R, Ballweg S, Levental I (2018). Cellular mechanisms of physicochemical membrane homeostasis. Curr Opin Cell Biol.

[102] Olzmann JA, Carvalho P (2019). Dynamics and functions of lipid droplets. Nat Rev Mol Cell Biol.

[103] Furse S, de Kroon AI (2015). Phosphatidylcholine's functions beyond that of a membrane brick. Mol Membr Biol.

[104] Long JZ, Cravatt BF (2011). The metabolic serine hydrolases and their functions in mammalian physiology and disease. Chem Rev.

[105] Joshi A, Shaikh M, Singh S, Rajendran A, Mhetre A, Kamat SS (2018). Biochemical characterization of the PHARC-associated serine hydrolase ABHD12 reveals its preference for very-long-chain lipids. J Biol Chem.

[106] Blankman JL, Simon GM, Cravatt BF (2007). A comprehensive profile of brain enzymes that hydrolyze the endocannabinoid 2-arachidonoylglycerol. Chem Biol.

[107] Blankman JL, Long JZ, Trauger SA, Siuzdak G, Cravatt BF (2013). ABHD12 controls brain lysophosphatidylserine pathways that are deregulated in a murine model of the neurodegenerative disease PHARC. Proc Natl Acad Sci U S A.

[108] Fiskerstrand T, H'Mida-Ben BD, Johansson S, M'Zahem A, Haukanes BI, Drouot N, Zimmermann J, Cole AJ (2010). Mutations in ABHD12 cause the neurodegenerative disease PHARC: An inborn error of endocannabinoid metabolism. Am J Hum Genet.

[109] Thimm A, Rahal A, Schoen U, Abicht A, Klebe S, Kleinschnitz C, Hagenacker T, Stettner M (2020). Genotype-phenotype correlation in a novel ABHD12 mutation underlying PHARC syndrome. J Peripher Nerv Syst.

[110] Eisenberger T, Slim R, Mansour A, Nauck M, Nurnberg G, Nurnberg P, Decker C, Dafinger C (2012). Targeted next-generation sequencing identifies a homozygous nonsense mutation in ABHD12, the gene underlying PHARC, in a family clinically diagnosed with Usher syndrome type 3. Orphanet J Rare Dis.

[111] Yoshimura H, Hashimoto T, Murata T, Fukushima K, Sugaya A, Nishio SY, Usami S (2015). Novel ABHD12 mutations in PHARC patients: the differential diagnosis of deaf-blindness. Ann Otol Rhinol Laryngol.

[112] Li T, Feng Y, Liu Y, He C, Liu J, Chen H, Deng Y, Li M (2019). A novel ABHD12 nonsense variant in Usher syndrome type 3 family with genotype-phenotype spectrum review. Gene.

[113] Singh S, Joshi A, Kamat SS (2020). Mapping the Neuroanatomy of ABHD16A, ABHD12, and Lysophosphatidylserines Provides New Insights into the Pathophysiology of the Human Neurological Disorder PHARC. Biochemistry-Us.

[114] Leishman E, Mackie K, Bradshaw HB (2019). Elevated Levels of Arachidonic Acid-Derived Lipids Including Prostaglandins and Endocannabinoids Are Present Throughout ABHD12 Knockout Brains: Novel Insights Into the Neurodegenerative Phenotype. Front Mol Neurosci.

[115] Savinainen JR, Kansanen E, Pantsar T, Navia-Paldanius D, Parkkari T, Lehtonen M, Laitinen T, Nevalainen T (2014). Robust hydrolysis of prostaglandin glycerol esters by human monoacylglycerol lipase (MAGL). Mol Pharmacol.

[116] Kavetsky L, Green KK, Boyle BR, Yousufzai F, Padron ZM, Melli SE, Kuhnel VL, Jackson HM (2019). Increased interactions and engulfment of dendrites by microglia precede Purkinje cell degeneration in a mouse model of Niemann Pick Type-C. Sci Rep.

[117] Safo PK, Regehr WG (2005). Endocannabinoids control the induction of cerebellar LTD. Neuron.

[118] Carey MR, Myoga MH, Mcdaniels KR, Marsicano G, Lutz B, Mackie K, Regehr WG (2011). Presynaptic CB1 receptors regulate synaptic plasticity at cerebellar parallel fiber synapses. J Neurophysiol.

[119] Kano M, Watanabe T (2017). Type-1 metabotropic glutamate receptor signaling in cerebellar Purkinje cells in health and disease. F1000Res.

[120] Tomas-Roig J, Agbemenyah HY, Celarain N, Quintana E, Ramio-Torrenta L, Havemann-Reinecke U (2020). Dose-dependent effect of cannabinoid WIN-55,212–2 on myelin repair following a demyelinating insult. Sci Rep.

[121] Chanda PK, Gao Y, Mark L, Btesh J, Strassle BW, Lu P, Piesla MJ, Zhang MY (2010). Monoacylglycerol lipase activity is a critical modulator of the tone and integrity of the endocannabinoid system. Mol Pharmacol.

[122] Makide K, Uwamizu A, Shinjo Y, Ishiguro J, Okutani M, Inoue A, Aoki J (2014). Novel lysophosphoplipid receptors: their structure and function. J Lipid Res.

[123] Yung YC, Stoddard NC, Chun J (2014). LPA receptor signaling: pharmacology, physiology, and pathophysiology. J Lipid Res.

[124] Santos-Nogueira E, Lopez-Serrano C, Hernandez J, Lago N, Astudillo AM, Balsinde J, Estivill-Torrus G, de Fonseca FR (2015). Activation of Lysophosphatidic Acid Receptor Type 1 Contributes to Pathophysiology of Spinal Cord Injury. J Neurosci.

[125] Liu X, Quan N (2018). Microglia and CNS Interleukin-1: Beyond Immunological Concepts. Front Neurol.

[126] Seppi D, Puthenparampil M, Federle L, Ruggero S, Toffanin E, Rinaldi F, Perini P, Gallo P (2014). Cerebrospinal fluid IL-1beta correlates with cortical pathology load in multiple sclerosis at clinical onset. J Neuroimmunol.

[127] Bozza PT, Viola JP (2010). Lipid droplets in inflammation and cancer. Prostaglandins Leukot Essent Fatty Acids.

[128] Chandak PG, Radovic B, Aflaki E, Kolb D, Buchebner M, Frohlich E, Magnes C, Sinner F (2010). Efficient phagocytosis requires triacylglycerol hydrolysis by adipose triglyceride lipase. J Biol Chem.

[129] Zoni V, Khaddaj R, Campomanes P, Thiam AR, Schneiter R, Vanni S (2020). Lipid Droplet Biogenesis is Driven by Liquid-Liquid Phase Separation.

[130] Dupont N, Chauhan S, Arko-Mensah J, Castillo EF, Masedunskas A, Weigert R, Robenek H, Proikas-Cezanne T (2014). Neutral lipid stores and lipase PNPLA5 contribute to autophagosome biogenesis. Curr Biol.

[131] Park SY, Yang JS, Li Z, Deng P, Zhu X, Young D, Ericsson M, Andringa R (2019). The late stage of COPI vesicle fission requires shorter forms of phosphatidic acid and diacylglycerol. Nat Commun.

[132] Yildiz Y, Matern H, Thompson B, Allegood JC, Warren RL, Ramirez DM, Hammer RE, Hamra FK (2006). Mutation of beta-glucosidase 2 causes glycolipid storage disease and impaired male fertility. J Clin Invest.

[133] Boot RG, Verhoek M, Donker-Koopman W, Strijland A, van Marle J, Overkleeft HS, Wennekes T, Aerts JM (2007). Identification of the non-lysosomal glucosylceramidase as beta-glucosidase 2. J Biol Chem.

[134] Korschen HG, Yildiz Y, Raju DN, Schonauer S, Bonigk W, Jansen V, Kremmer E, Kaupp UB (2013). The non-lysosomal beta-glucosidase GBA2 is a non-integral membrane-associated protein at the endoplasmic reticulum (ER) and Golgi. J Biol Chem.

[135] Marques AR, Aten J, Ottenhoff R, van Roomen CP, Herrera MD, Claessen N, Vinueza VM, Zhou K (2015). Reducing GBA2 Activity Ameliorates Neuropathology in Niemann-Pick Type C Mice. PLoS ONE.

[136] Herrera MCD, Kallemeijn WW, Marques AR, Orre M, Ottenhoff R, van Roomen C, Foppen E, Renner MC (2015). Visualization of Active Glucocerebrosidase in Rodent Brain with High Spatial Resolution following In Situ Labeling with Fluorescent Activity Based Probes. PLoS ONE.

[137] Woeste MA, Stern S, Raju DN, Grahn E, Dittmann D, Gutbrod K, Dormann P, Hansen JN (2019). Species-specific differences in nonlysosomal glucosylceramidase GBA2 function underlie locomotor dysfunction arising from loss-of-function mutations. J Biol Chem.

[138] Martin E, Schule R, Smets K, Rastetter A, Boukhris A, Loureiro JL, Gonzalez MA, Mundwiller E (2013). Loss of function of glucocerebrosidase GBA2 is responsible for motor neuron defects in hereditary spastic paraplegia. Am J Hum Genet.

[139] Hammer MB, Eleuch-Fayache G, Schottlaender LV, Nehdi H, Gibbs JR, Arepalli SK, Chong SB, Hernandez DG (2013). Mutations in GBA2 cause autosomal-recessive cerebellar ataxia with spasticity. Am J Hum Genet.

[140] Sultana S, Reichbauer J, Schule R, Mochel F, Synofzik M, van der Spoel AC (2015). Lack of enzyme activity in GBA2 mutants associated with hereditary spastic paraplegia/cerebellar ataxia (SPG46). Biochem Biophys Res Commun.

[141] Spagnoli C, Schiavoni S, Rizzi S, Salerno GG, Frattini D, Fusco C (2020). New biallelic GBA2 variant in a patient with SPG46. Clin Neurol Neurosurg.

[142] Haugarvoll K, Johansson S, Rodriguez CE, Boman H, Haukanes BI, Bruland O, Roque F, Jonassen I (2017). GBA2 Mutations Cause a Marinesco-Sjogren-Like Syndrome: Genetic and Biochemical Studies. PLoS ONE.

[143] Walden CM, Sandhoff R, Chuang CC, Yildiz Y, Butters TD, Dwek RA, Platt FM, van der Spoel AC (2007). Accumulation of glucosylceramide in murine testis, caused by inhibition of beta-glucosidase 2: implications for spermatogenesis. J Biol Chem.

[144] Gonzalez-Carmona MA, Sandhoff R, Tacke F, Vogt A, Weber S, Canbay AE, Rogler G, Sauerbruch T (2012). Beta-glucosidase 2 knockout mice with increased glucosylceramide show impaired liver regeneration. Liver Int.

[145] Raju D, Schonauer S, Hamzeh H, Flynn KC, Bradke F, Vom DK, Dormann P, Yildiz Y (2015). Accumulation of glucosylceramide in the absence of the beta-glucosidase GBA2 alters cytoskeletal dynamics. Plos Genet.

[146] Wheeler S, Haberkant P, Bhardwaj M, Tongue P, Ferraz MJ, Halter D, Sprong H, Schmid R (2019). Cytosolic glucosylceramide regulates endolysosomal function in Niemann-Pick type C disease. Neurobiol Dis.

[147] Burke DG, Rahim AA, Waddington SN, Karlsson S, Enquist I, Bhatia K, Mehta A, Vellodi A (2013). Increased glucocerebrosidase (GBA) 2 activity in GBA1 deficient mice brains and in Gaucher leucocytes. J Inherit Metab Dis.

[148] Yildiz Y, Hoffmann P, Vom DS, Breiden B, Sandhoff R, Niederau C, Horwitz M, Karlsson S (2013). Functional and genetic characterization of the non-lysosomal glucosylceramidase 2 as a modifier for Gaucher disease. Orphanet J Rare Dis.

[149] Huebecker M, Moloney EB, van der Spoel AC, Priestman DA, Isacson O, Hallett PJ, Platt FM (2019). Reduced sphingolipid hydrolase activities, substrate accumulation and ganglioside decline in Parkinson's disease. Mol Neurodegener.

[150] Franco R, Sanchez-Arias JA, Navarro G, Lanciego JL (2018). Glucocerebrosidase Mutations and Synucleinopathies. Potential Role of Sterylglucosides and Relevance of Studying Both GBA1 and GBA2 Genes. Front Neuroanat.

[151] Hanada K, Kumagai K, Yasuda S, Miura Y, Kawano M, Fukasawa M, Nishijima M (2003). Molecular machinery for non-vesicular trafficking of ceramide. Nature.

[152] D'Angelo G, Polishchuk E, Di Tullio G, Santoro M, Di Campli A, Godi A, West G, Bielawski J (2007). Glycosphingolipid synthesis requires FAPP2 transfer of glucosylceramide. Nature.

[153] Halter D, Neumann S, van Dijk SM, Wolthoorn J, de Maziere AM, Vieira OV, Mattjus P, Klumperman J (2007). Pre- and post-Golgi translocation of glucosylceramide in glycosphingolipid synthesis. J Cell Biol.

[154] Nakamura S, Oba M, Suzuki M, Takahashi A, Yamamuro T, Fujiwara M, Ikenaka K, Minami S (2019). Suppression of autophagic activity by Rubicon is a signature of aging. Nat Commun.

[155] Jakobsson A, Westerberg R, Jacobsson A (2006). Fatty acid elongases in mammals: their regulation and roles in metabolism. Prog Lipid Res.

[156] Ohno Y, Suto S, Yamanaka M, Mizutani Y, Mitsutake S, Igarashi Y, Sassa T, Kihara A (2010). ELOVL1 production of C24 acyl-CoAs is linked to C24 sphingolipid synthesis. Proc Natl Acad Sci U S A.

[157] Sassa T, Kihara A (2014). Metabolism of very long-chain Fatty acids: genes and pathophysiology. Biomol Ther (Seoul).

[158] Kihara A (2012). Very long-chain fatty acids: elongation, physiology and related disorders. J Biochem.

[159] Agbaga MP, Brush RS, Mandal MN, Henry K, Elliott MH, Anderson RE (2008). Role of Stargardt-3 macular dystrophy protein (ELOVL4) in the biosynthesis of very long chain fatty acids. Proc Natl Acad Sci U S A.

[160] Moon YA, Hammer RE, Horton JD (2009). Deletion of ELOVL5 leads to fatty liver through activation of SREBP-1c in mice. J Lipid Res.

[161] Di Gregorio E, Borroni B, Giorgio E, Lacerenza D, Ferrero M, Lo BN, Ragusa N, Mancini C (2014). ELOVL5 mutations cause spinocerebellar ataxia 38. Am J Hum Genet.

[162] Agbaga MP, Merriman DK, Brush RS, Lydic TA, Conley SM, Naash MI, Jackson S, Woods AS (2018). Differential composition of DHA and very-long-chain PUFAs in rod and cone photoreceptors. J Lipid Res.

[163] Hoxha E, Gabriele R, Balbo I, Ravera F, Masante L, Zambelli V, Albergo C, Mitro N (2017). Motor Deficits and Cerebellar Atrophy in Elovl5 Knock Out Mice. Front Cell Neurosci.

[164] Sherry DM, Hopiavuori BR, Stiles MA, Rahman NS, Ozan KG, Deak F, Agbaga MP, Anderson RE (2017). Distribution of ELOVL4 in the Developing and Adult Mouse Brain. Front Neuroanat.

[165] Cadieux-Dion M, Turcotte-Gauthier M, Noreau A, Martin C, Meloche C, Gravel M, Drouin CA, Rouleau GA (2014). Expanding the clinical phenotype associated with ELOVL4 mutation: study of a large French-Canadian family with autosomal dominant spinocerebellar ataxia and erythrokeratodermia. Jama Neurol.

[166] Ozaki K, Doi H, Mitsui J, Sato N, Iikuni Y, Majima T, Yamane K, Irioka T (2015). A Novel Mutation in ELOVL4 Leading to Spinocerebellar Ataxia (SCA) With the Hot Cross Bun Sign but Lacking Erythrokeratodermia: A Broadened Spectrum of SCA34. Jama Neurol.

[167] Ozaki K, Ansai A, Nobuhara K, Araki T, Kubodera T, Ishii T, Higashi M, Sato N (2019). Prevalence and clinicoradiological features of spinocerebellar ataxia type 34 in a Japanese ataxia cohort. Parkinsonism Relat Disord.

[168] Beaudin M, Sellami L, Martel C, Touzel-Deschenes L, Houle G, Martineau L, Lacroix K, Lavallee A (2020). Characterization of the phenotype with cognitive impairment and protein mislocalization in SCA34. Neurol Genet.

[169] Borroni B, Di Gregorio E, Orsi L, Vaula G, Costanzi C, Tempia F, Mitro N, Caruso D (2016). Clinical and neuroradiological features of spinocerebellar ataxia 38 (SCA38). Parkinsonism Relat Disord.

[170] Hopiavuori BR, Anderson RE, Agbaga MP (2019). ELOVL4: Very long-chain fatty acids serve an eclectic role in mammalian health and function. Prog Retin Eye Res.

[171] Deak F, Anderson RE, Fessler JL, Sherry DM (2019). Novel Cellular Functions of Very Long Chain-Fatty Acids: Insight From ELOVL4 Mutations. Front Cell Neurosci.

[172] Wakashima T, Abe K, Kihara A (2014). Dual functions of the trans-2-enoyl-CoA reductase TER in the sphingosine 1-phosphate metabolic pathway and in fatty acid elongation. J Biol Chem.

[173] Agbaga MP (2016). Different Mutations in ELOVL4 Affect Very Long Chain Fatty Acid Biosynthesis to Cause Variable Neurological Disorders in Humans. Adv Exp Med Biol.

[174] Ferrer I, Kapfhammer JP, Hindelang C, Kemp S, Troffer-Charlier N, Broccoli V, Callyzot N, Mooyer P (2005). Inactivation of the peroxisomal ABCD2 transporter in the mouse leads to late-onset ataxia involving mitochondria, Golgi and endoplasmic reticulum damage. Hum Mol Genet.

[175] Mohamed B, Mazeaud C, Baril M, Poirier D, Sow AA, Chatel-Chaix L, Titorenko V, Lamarre D (2020). Very-long-chain fatty acid metabolic capacity of 17-beta-hydroxysteroid dehydrogenase type 12 (HSD17B12) promotes replication of hepatitis C virus and related flaviviruses. Sci Rep.

[176] Jarius S, Wandinger KP, Horn S, Heuer H, Wildemann B (2010). A new Purkinje cell antibody (anti-Ca) associated with subacute cerebellar ataxia: immunological characterization. J Neuroinflammation.

[177] Lucken-Ardjomande HS, Vallis Y, Jolin HE, Mckenzie AN, Mcmahon HT (2014). GRAF1a is a brain-specific protein that promotes lipid droplet clustering and growth, and is enriched at lipid droplet junctions. J Cell Sci.

[178] Logan S, Agbaga MP, Chan MD, Kabir N, Mandal NA, Brush RS, Anderson RE (2013). Deciphering mutant ELOVL4 activity in autosomal-dominant Stargardt macular dystrophy. Proc Natl Acad Sci U S A.

[179] Esteve-Rudd J, Hazim RA, Diemer T, Paniagua AE, Volland S, Umapathy A, Williams DS (2018). Defective phagosome motility and degradation in cell nonautonomous RPE pathogenesis of a dominant macular degeneration. Proc Natl Acad Sci U S A.

[180] Nagase T, Seki N, Ishikawa K, Ohira M, Kawarabayasi Y, Ohara O, Tanaka A, Kotani H et al (1996) Prediction of the coding sequences of unidentified human genes. VI. The coding sequences of 80 new genes (KIAA0201-KIAA0280) deduced by analysis of cDNA clones from cell line KG-1 and brain. Dna Res 3(5):321–329, 341–354 10.1093/dnares/3.5.321 10.1093/dnares/3.5.321 9039502

[181] Zhong Y, Wang QJ, Li X, Yan Y, Backer JM, Chait BT, Heintz N, Yue Z (2009). Distinct regulation of autophagic activity by Atg14L and Rubicon associated with Beclin 1-phosphatidylinositol-3-kinase complex. Nat Cell Biol.

[182] Matsunaga K, Noda T, Yoshimori T (2009). Binding Rubicon to cross the Rubicon. Autophagy.

[183] Martinez J (2018). LAP it up, fuzz ball: a short history of LC3-associated phagocytosis. Curr Opin Immunol.

[184] Sun Q, Westphal W, Wong KN, Tan I, Zhong Q (2010). Rubicon controls endosome maturation as a Rab7 effector. Proc Natl Acad Sci U S A.

[185] Yang CS, Lee JS, Rodgers M, Min CK, Lee JY, Kim HJ, Lee KH, Kim CJ (2012). Autophagy protein Rubicon mediates phagocytic NADPH oxidase activation in response to microbial infection or TLR stimulation. Cell Host Microbe.

[186] Yang CS, Rodgers M, Min CK, Lee JS, Kingeter L, Lee JY, Jong A, Kramnik I (2012). The autophagy regulator Rubicon is a feedback inhibitor of CARD9-mediated host innate immunity. Cell Host Microbe.

[187] Sun Q, Zhang J, Fan W, Wong KN, Ding X, Chen S, Zhong Q (2011). The RUN domain of rubicon is important for hVps34 binding, lipid kinase inhibition, and autophagy suppression. J Biol Chem.

[188] Martinez J, Malireddi RK, Lu Q, Cunha LD, Pelletier S, Gingras S, Orchard R, Guan JL (2015). Molecular characterization of LC3-associated phagocytosis reveals distinct roles for Rubicon, NOX2 and autophagy proteins. Nat Cell Biol.

[189] Wong SW, Sil P, Martinez J (2018). Rubicon: LC3-associated phagocytosis and beyond. Febs J.

[190] Assoum M, Salih MA, Drouot N, H'Mida-Ben BD, Lagier-Tourenne C, Aldrees A, Elmalik SA, Ahmed TS (2010). Rundataxin, a novel protein with RUN and diacylglycerol binding domains, is mutant in a new recessive ataxia. Brain.

[191] Seidahmed MZ, Hamad MH, Albakheet A, Elmalik SA, Aldrees A, Al-Sufayan J, Alorainy I, Ghozzi IM (2020). Ancient founder mutation in RUBCN: a second unrelated family confirms Salih ataxia (SCAR15). Bmc Neurol.

[192] Assoum M, Salih MA, Drouot N, Hnia K, Martelli A, Koenig M (2013). The Salih ataxia mutation impairs Rubicon endosomal localization. Cerebellum.

[193] Tanaka S, Hikita H, Tatsumi T, Sakamori R, Nozaki Y, Sakane S, Shiode Y, Nakabori T (2016). Rubicon inhibits autophagy and accelerates hepatocyte apoptosis and lipid accumulation in nonalcoholic fatty liver disease in mice. Hepatology.

[194] Gaullier JM, Simonsen A, D'Arrigo A, Bremnes B, Stenmark H, Aasland R (1998). FYVE fingers bind PtdIns(3)P. Nature.

[195] Leevers SJ, Vanhaesebroeck B, Waterfield MD (1999). Signalling through phosphoinositide 3-kinases: the lipids take centre stage. Curr Opin Cell Biol.

[196] Tabata K, Matsunaga K, Sakane A, Sasaki T, Noda T, Yoshimori T (2010). Rubicon and PLEKHM1 negatively regulate the endocytic/autophagic pathway via a novel Rab7-binding domain. Mol Biol Cell.

[197] Liu K, Jian Y, Sun X, Yang C, Gao Z, Zhang Z, Liu X, Li Y (2016). Negative regulation of phosphatidylinositol 3-phosphate levels in early-to-late endosome conversion. J Cell Biol.

[198] Rapiteanu R, Davis LJ, Williamson JC, Timms RT, Paul LJ, Lehner PJ (2016). A Genetic Screen Identifies a Critical Role for the WDR81-WDR91 Complex in the Trafficking and Degradation of Tetherin. Traffic.

[199] Baba T, Toth DJ, Sengupta N, Kim YJ, Balla T (2019). Phosphatidylinositol 4,5-bisphosphate controls Rab7 and PLEKHM1 membrane cycling during autophagosome-lysosome fusion. Embo J.

[200] Meneses-Salas E, Garcia-Melero A, Kanerva K, Blanco-Munoz P, Morales-Paytuvi F, Bonjoch J, Casas J, Egert A (2020). Annexin A6 modulates TBC1D15/Rab7/StARD3 axis to control endosomal cholesterol export in NPC1 cells. Cell Mol Life Sci.

[201] Mitchell AG, Martin CE (1997). Fah1p, a Saccharomyces cerevisiae cytochrome b5 fusion protein, and its Arabidopsis thaliana homolog that lacks the cytochrome b5 domain both function in the alpha-hydroxylation of sphingolipid-associated very long chain fatty acids. J Biol Chem.

[202] Alderson NL, Rembiesa BM, Walla MD, Bielawska A, Bielawski J, Hama H (2004). The human FA2H gene encodes a fatty acid 2-hydroxylase. J Biol Chem.

[203] Eckhardt M, Yaghootfam A, Fewou SN, Zoller I, Gieselmann V (2005). A mammalian fatty acid hydroxylase responsible for the formation of alpha-hydroxylated galactosylceramide in myelin. Biochem J.

[204] Zhu G, Koszelak-Rosenblum M, Connelly SM, Dumont ME, Malkowski MG (2015). The Crystal Structure of an Integral Membrane Fatty Acid alpha-Hydroxylase. J Biol Chem.

[205] Guo L, Zhang X, Zhou D, Okunade AL, Su X (2012). Stereospecificity of fatty acid 2-hydroxylase and differential functions of 2-hydroxy fatty acid enantiomers. J Lipid Res.

[206] Oh CS, Toke DA, Mandala S, Martin CE (1997). ELO2 and ELO3, homologues of the Saccharomyces cerevisiae ELO1 gene, function in fatty acid elongation and are required for sphingolipid formation. J Biol Chem.

[207] Edvardson S, Hama H, Shaag A, Gomori JM, Berger I, Soffer D, Korman SH, Taustein I (2008). Mutations in the fatty acid 2-hydroxylase gene are associated with leukodystrophy with spastic paraparesis and dystonia. Am J Hum Genet.

[208] Dick KJ, Eckhardt M, Paisan-Ruiz C, Alshehhi AA, Proukakis C, Sibtain NA, Maier H, Sharifi R (2010). Mutation of FA2H underlies a complicated form of hereditary spastic paraplegia (SPG35). Hum Mutat.

[209] Marelli C, Salih MA, Nguyen K, Mallaret M, Leboucq N, Hassan HH, Drouot N, Labauge P (2015). Cerebral Iron Accumulation Is Not a Major Feature of FA2H/SPG35. Mov Disord Clin Pract.

[210] Rattay TW, Lindig T, Baets J, Smets K, Deconinck T, Sohn AS, Hortnagel K, Eckstein KN (2019). FAHN/SPG35: a narrow phenotypic spectrum across disease classifications. Brain.

[211] Synofzik M, Schule R (2017). Overcoming the divide between ataxias and spastic paraplegias: Shared phenotypes, genes, and pathways. Mov Disord.

[212] Potter KA, Kern MJ, Fullbright G, Bielawski J, Scherer SS, Yum SW, Li JJ, Cheng H (2011). Central nervous system dysfunction in a mouse model of FA2H deficiency. Glia.

[213] Zoller I, Meixner M, Hartmann D, Bussow H, Meyer R, Gieselmann V, Eckhardt M (2008). Absence of 2-hydroxylated sphingolipids is compatible with normal neural development but causes late-onset axon and myelin sheath degeneration. J Neurosci.

[214] Kota V, Hama H (2014). 2'-Hydroxy ceramide in membrane homeostasis and cell signaling. Adv Biol Regul.

[215] Hardt R, Winter D, Gieselmann V, Eckhardt M (2018). Identification of progesterone receptor membrane component-1 as an interaction partner and possible regulator of fatty acid 2-hydroxylase. Biochem J.

[216] Piel RR, Shiferaw MT, Vashisht AA, Marcero JR, Praissman JL, Phillips JD, Wohlschlegel JA, Medlock AE (2016). A Novel Role for Progesterone Receptor Membrane Component 1 (PGRMC1): A Partner and Regulator of Ferrochelatase. Biochemistry-Us.

[217] Mifsud W, Bateman A (2002). Membrane-bound progesterone receptors contain a cytochrome b5-like ligand-binding domain. Genome Biol.

[218] Hughes AL, Powell DW, Bard M, Eckstein J, Barbuch R, Link AJ, Espenshade PJ (2007). Dap1/PGRMC1 binds and regulates cytochrome P450 enzymes. Cell Metab.

[219] Suchanek M, Radzikowska A, Thiele C (2005). Photo-leucine and photo-methionine allow identification of protein-protein interactions in living cells. Nat Methods.

[220] Chuang SS, Helvig C, Taimi M, Ramshaw HA, Collop AH, Amad M, White JA, Petkovich M (2004). CYP2U1, a novel human thymus- and brain-specific cytochrome P450, catalyzes omega- and (omega-1)-hydroxylation of fatty acids. J Biol Chem.

[221] Dhers L, Ducassou L, Boucher JL, Mansuy D (2017). Cytochrome P450 2U1, a very peculiar member of the human P450s family. Cell Mol Life Sci.

[222] Okita RT, Okita JR (2001). Cytochrome P450 4A fatty acid omega hydroxylases. Curr Drug Metab.

[223] Fekry MI, Xiao Y, Berg JZ, Guengerich FP (2019). A Role for the Orphan Human Cytochrome P450 2S1 in Polyunsaturated Fatty Acid omega-1 Hydroxylation Using an Untargeted Metabolomic Approach. Drug Metab Dispos.

[224] Li Y, Wang C, Huang Y, Fu R, Zheng H, Zhu Y, Shi X, Padakanti PK (2018). C. Elegans Fatty Acid Two-Hydroxylase Regulates Intestinal Homeostasis by Affecting Heptadecenoic Acid Production. Cell Physiol Biochem.

[225] Cantagrel V, Lefeber DJ, Ng BG, Guan Z, Silhavy JL, Bielas SL, Lehle L, Hombauer H (2010). SRD5A3 is required for converting polyprenol to dolichol and is mutated in a congenital glycosylation disorder. Cell.

[226] Wheeler PG, Ng BG, Sanford L, Sutton VR, Bartholomew DW, Pastore MT, Bamshad MJ, Kircher M (2016). SRD5A3-CDG: Expanding the phenotype of a congenital disorder of glycosylation with emphasis on adult onset features. Am J Med Genet A.

[227] Morava E, Wevers RA, Cantagrel V, Hoefsloot LH, Al-Gazali L, Schoots J, van Rooij A, Huijben K (2010). A novel cerebello-ocular syndrome with abnormal glycosylation due to abnormalities in dolichol metabolism. Brain.

[228] Kasapkara CS, Tumer L, Ezgu FS, Hasanoglu A, Race V, Matthijs G, Jaeken J (2012). SRD5A3-CDG: a patient with a novel mutation. Eur J Paediatr Neurol.

[229] Medina-Cano D, Ucuncu E, Nguyen LS, Nicouleau M, Lipecka J, Bizot JC, Thiel C, Foulquier F et al (2018) High N-glycan multiplicity is critical for neuronal adhesion and sensitizes the developing cerebellum to N-glycosylation defect. Elife 710.7554/eLife.38309 10.7554/eLife.38309 PMC6185108 30311906

[230] Coleman JA, Molday RS (2011). Critical role of the beta-subunit CDC50A in the stable expression, assembly, subcellular localization, and lipid transport activity of the P4-ATPase ATP8A2. J Biol Chem.

[231] Zhao Y, Ren J, Harlos K, Stuart DI (2016). Structure of glycosylated NPC1 luminal domain C reveals insights into NPC2 and Ebola virus interactions. Febs Lett.

[232] Zhao JJ, Halvardson J, Knaus A, Georgii-Hemming P, Baeck P, Krawitz PM, Thuresson AC, Feuk L (2017). Reduced cell surface levels of GPI-linked markers in a new case with PIGG loss of function. Hum Mutat.

[233] Lukacs M, Blizzard LE, Stottmann RW (2020). CNS glycosylphosphatidylinositol deficiency results in delayed white matter development, ataxia and premature death in a novel mouse model. Hum Mol Genet.

[234] Nguyen T, Murakami Y, Sheridan E, Ehresmann S, Rousseau J, St-Denis A, Chai G, Ajeawung NF (2017). Mutations in GPAA1, Encoding a GPI Transamidase Complex Protein, Cause Developmental Delay, Epilepsy, Cerebellar Atrophy, and Osteopenia. Am J Hum Genet.

[235] Nguyen T, Murakami Y, Mobilio S, Niceta M, Zampino G, Philippe C, Moutton S, Zaki MS (2020). Bi-allelic Variants in the GPI Transamidase Subunit PIGK Cause a Neurodevelopmental Syndrome with Hypotonia, Cerebellar Atrophy, and Epilepsy. Am J Hum Genet.

[236] Ohba C, Okamoto N, Murakami Y, Suzuki Y, Tsurusaki Y, Nakashima M, Miyake N, Tanaka F (2014). PIGN mutations cause congenital anomalies, developmental delay, hypotonia, epilepsy, and progressive cerebellar atrophy. Neurogenetics.

[237] Li J, Deffieu MS, Lee PL, Saha P, Pfeffer SR (2015). Glycosylation inhibition reduces cholesterol accumulation in NPC1 protein-deficient cells. Proc Natl Acad Sci U S A.

[238] Sahu SK, Gummadi SN, Manoj N, Aradhyam GK (2007). Phospholipid scramblases: an overview. Arch Biochem Biophys.

[239] Hankins HM, Baldridge RD, Xu P, Graham TR (2015). Role of flippases, scramblases and transfer proteins in phosphatidylserine subcellular distribution. Traffic.

[240] Montigny C, Lyons J, Champeil P, Nissen P (1861). Lenoir G (2016) On the molecular mechanism of flippase- and scramblase-mediated phospholipid transport. Biochim Biophys Acta.

[241] Pedemonte N, Galietta LJ (2014). Structure and function of TMEM16 proteins (anoctamins). Physiol Rev.

[242] Suzuki J, Fujii T, Imao T, Ishihara K, Kuba H, Nagata S (2013). Calcium-dependent phospholipid scramblase activity of TMEM16 protein family members. J Biol Chem.

[243] Tian Y, Schreiber R, Kunzelmann K (2012). Anoctamins are a family of Ca2+-activated Cl- channels. J Cell Sci.

[244] Duran C, Hartzell HC (2011). Physiological roles and diseases of Tmem16/Anoctamin proteins: are they all chloride channels?. Acta Pharmacol Sin.

[245] Whitlock JM, Hartzell HC (2017). Anoctamins/TMEM16 Proteins: Chloride Channels Flirting with Lipids and Extracellular Vesicles. Annu Rev Physiol.

[246] Bushell SR, Pike A, Falzone ME, Rorsman N, Ta CM, Corey RA, Newport TD, Christianson JC (2019). The structural basis of lipid scrambling and inactivation in the endoplasmic reticulum scramblase TMEM16K. Nat Commun.

[247] Tsuji T, Cheng J, Tatematsu T, Ebata A, Kamikawa H, Fujita A, Gyobu S, Segawa K (2019). Predominant localization of phosphatidylserine at the cytoplasmic leaflet of the ER, and its TMEM16K-dependent redistribution. Proc Natl Acad Sci U S A.

[248] Cabrita I, Benedetto R, Fonseca A, Wanitchakool P, Sirianant L, Skryabin BV, Schenk LK, Pavenstadt H (2017). Differential effects of anoctamins on intracellular calcium signals. Faseb J.

[249] Vermeer S, Hoischen A, Meijer RP, Gilissen C, Neveling K, Wieskamp N, de Brouwer A, Koenig M (2010). Targeted next-generation sequencing of a 12.5 Mb homozygous region reveals ANO10 mutations in patients with autosomal-recessive cerebellar ataxia. Am J Hum Genet.

[250] Nanetti L, Sarto E, Castaldo A, Magri S, Mongelli A, Rossi SD, Canafoglia L, Grisoli M (2019). ANO10 mutational screening in recessive ataxia: genetic findings and refinement of the clinical phenotype. J Neurol.

[251] Benarroch EE (2017). Anoctamins (TMEM16 proteins): Functions and involvement in neurologic disease. Neurology.

[252] Rossi M, Anheim M, Durr A, Klein C, Koenig M, Synofzik M, Marras C, van de Warrenburg BP (2018). The genetic nomenclature of recessive cerebellar ataxias. Mov Disord.

[253] Ballas LM, Bell RM (1980). Topography of phosphatidylcholine, phosphatidylethanolamine and triacylgycerol biosynthetic enzymes in rat liver microsomes. Biochim Biophys Acta.

[254] Ballas LM, Bell RM (1981). Topography of glycerolipid synthetic enzymes. Synthesis of phosphatidylserine, phosphatidylinositol and glycerolipid intermediates occurs on the cytoplasmic surface of rat liver microsomal vesicles. Biochim Biophys Acta.

[255] Devaux PF, Herrmann A, Ohlwein N, Kozlov MM (2008). How lipid flippases can modulate membrane structure. Biochim Biophys Acta.

[256] Marx U, Lassmann G, Holzhutter HG, Wustner D, Muller P, Hohlig A, Kubelt J, Herrmann A (2000). Rapid flip-flop of phospholipids in endoplasmic reticulum membranes studied by a stopped-flow approach. Biophys J.

[257] Gummadi SN, Kumar KS (2005). The mystery of phospholipid flip-flop in biogenic membranes. Cell Mol Biol Lett.

[258] Goren MA, Morizumi T, Menon I, Joseph JS, Dittman JS, Cherezov V, Stevens RC, Ernst OP (2014). Constitutive phospholipid scramblase activity of a G protein-coupled receptor. Nat Commun.

[259] Reggio PH (2018). GPCRs Moonlighting as Scramblases: Mechanism Revealed. Structure.

[260] Wang L, Iwasaki Y, Andra KK, Pandey K, Menon AK, Butikofer P (2018). Scrambling of natural and fluorescently tagged phosphatidylinositol by reconstituted G protein-coupled receptor and TMEM16 scramblases. J Biol Chem.

[261] Pomorski TG, Menon AK (2016). Lipid somersaults: Uncovering the mechanisms of protein-mediated lipid flipping. Prog Lipid Res.

[262] Fu S, Yang L, Li P, Hofmann O, Dicker L, Hide W, Lin X, Watkins SM (2011). Aberrant lipid metabolism disrupts calcium homeostasis causing liver endoplasmic reticulum stress in obesity. Nature.

[263] Karagas NE, Venkatachalam K (2019) Roles for the Endoplasmic Reticulum in Regulation of Neuronal Calcium Homeostasis. Cells-Basel 8(10). 10.3390/cells8101232 10.3390/cells8101232 PMC6829861 31658749

[264] Yu Y, Xia X, Li H, Zhang Y, Zhou X, Jiang H (2019). A new rhodopsin R135W mutation induces endoplasmic reticulum stress and apoptosis in retinal pigment epithelial cells. J Cell Physiol.

[265] Dalal S, Foster CR, Das BC, Singh M, Singh K (2012). Beta-adrenergic receptor stimulation induces endoplasmic reticulum stress in adult cardiac myocytes: role in apoptosis. Mol Cell Biochem.

[266] Arasi FP, Shahrestanaki MK, Aghaei M (2019). A2a adenosine receptor agonist improves endoplasmic reticulum stress in MIN6 cell line through protein kinase A/ protein kinase B/ Cyclic adenosine monophosphate response element-binding protein/ and Growth Arrest And DNA-Damage-Inducible 34/ eukaryotic Initiation Factor 2alpha pathways. J Cell Physiol.

[267] Hammer C, Wanitchakool P, Sirianant L, Papiol S, Monnheimer M, Faria D, Ousingsawat J, Schramek N (2015). A Coding Variant of ANO10, Affecting Volume Regulation of Macrophages, Is Associated with Borrelia Seropositivity. Mol Med.

[268] Wanitchakool P, Ousingsawat J, Sirianant L, Cabrita I, Faria D, Schreiber R, Kunzelmann K (2017). Cellular defects by deletion of ANO10 are due to deregulated local calcium signaling. Cell Signal.

[269] Ben MK, Ajjaji D, Chorlay A, Vanni S, Foret L, Thiam AR (2017). ER Membrane Phospholipids and Surface Tension Control Cellular Lipid Droplet Formation. Dev Cell.

[270] Chorlay A, Thiam AR (2018). An Asymmetry in Monolayer Tension Regulates Lipid Droplet Budding Direction. Biophys J.

[271] Bai Y, Meng L, Han L, Jia Y, Zhao Y, Gao H, Kang R, Wang X (2019). Lipid storage and lipophagy regulates ferroptosis. Biochem Biophys Res Commun.

[272] Katoh Y, Katoh M (2004). Identification and characterization of CDC50A, CDC50B and CDC50C genes in silico. Oncol Rep.

[273] van der Velden LM, Wichers CG, van Breevoort AE, Coleman JA, Molday RS, Berger R, Klomp LW, van de Graaf SF (2010). Heteromeric interactions required for abundance and subcellular localization of human CDC50 proteins and class 1 P4-ATPases. J Biol Chem.

[274] Bryde S, Hennrich H, Verhulst PM, Devaux PF, Lenoir G, Holthuis JC (2010). CDC50 proteins are critical components of the human class-1 P4-ATPase transport machinery. J Biol Chem.

[275] van der Mark VA, Elferink RP, Paulusma CC (2013). P4 ATPases: flippases in health and disease. Int J Mol Sci.

[276] Andersen JP, Vestergaard AL, Mikkelsen SA, Mogensen LS, Chalat M, Molday RS (2016). P4-ATPases as Phospholipid Flippases-Structure, Function, and Enigmas. Front Physiol.

[277] Baldridge RD, Graham TR (2012). Identification of residues defining phospholipid flippase substrate specificity of type IV P-type ATPases. Proc Natl Acad Sci U S A.

[278] Baldridge RD, Graham TR (2013). Two-gate mechanism for phospholipid selection and transport by type IV P-type ATPases. Proc Natl Acad Sci U S A.

[279] Baldridge RD, Xu P, Graham TR (2013). Type IV P-type ATPases distinguish mono- versus diacyl phosphatidylserine using a cytofacial exit gate in the membrane domain. J Biol Chem.

[280] Coleman JA, Kwok MC, Molday RS (2009). Localization, purification, and functional reconstitution of the P4-ATPase Atp8a2, a phosphatidylserine flippase in photoreceptor disc membranes. J Biol Chem.

[281] Tadini-Buoninsegni F, Mikkelsen SA, Mogensen LS, Molday RS, Andersen JP (2019). Phosphatidylserine flipping by the P4-ATPase ATP8A2 is electrogenic. Proc Natl Acad Sci U S A.

[282] Yang Y, Sun K, Liu W, Zhang L, Peng K, Zhang S, Li S, Yang M (2018). Disruption of Tmem30a results in cerebellar ataxia and degeneration of Purkinje cells. Cell Death Dis.

[283] Cacciagli P, Haddad MR, Mignon-Ravix C, El-Waly B, Moncla A, Missirian C, Chabrol B, Villard L (2010). Disruption of the ATP8A2 gene in a patient with a t(10;13) de novo balanced translocation and a severe neurological phenotype. Eur J Hum Genet.

[284] Zhu X, Libby RT, de Vries WN, Smith RS, Wright DL, Bronson RT, Seburn KL, John SW (2012). Mutations in a P-type ATPase gene cause axonal degeneration. Plos Genet.

[285] Onat OE, Gulsuner S, Bilguvar K, Nazli BA, Topaloglu H, Tan M, Tan U, Gunel M (2013). Missense mutation in the ATPase, aminophospholipid transporter protein ATP8A2 is associated with cerebellar atrophy and quadrupedal locomotion. Eur J Hum Genet.

[286] Kodigepalli KM, Bowers K, Sharp A, Nanjundan M (2015). Roles and regulation of phospholipid scramblases. Febs Lett.

[287] Castegna A, Lauderback CM, Mohmmad-Abdul H, Butterfield DA (2004). Modulation of phospholipid asymmetry in synaptosomal membranes by the lipid peroxidation products, 4-hydroxynonenal and acrolein: implications for Alzheimer's disease. Brain Res.

[288] Paulusma CC, Folmer DE, Ho-Mok KS, de Waart DR, Hilarius PM, Verhoeven AJ, Oude ER (2008). ATP8B1 requires an accessory protein for endoplasmic reticulum exit and plasma membrane lipid flippase activity. Hepatology.

[289] Jackson CL, Walch L, Verbavatz JM (2016). Lipids and Their Trafficking: An Integral Part of Cellular Organization. Dev Cell.

[290] Su X, Abumrad NA (2009). Cellular fatty acid uptake: a pathway under construction. Trends Endocrinol Metab.

[291] Afonso MS, Machado RM, Lavrador MS, Quintao E, Moore KJ, Lottenberg AM (2018) Molecular Pathways Underlying Cholesterol Homeostasis. Nutrients 10(6). 10.3390/nu10060760 10.3390/nu10060760 PMC6024674 29899250

[292] Prinz WA (2010). Lipid trafficking sans vesicles: where, why, how?. Cell.

[293] Balla T, Kim YJ, Alvarez-Prats A, Pemberton J (2019). Lipid Dynamics at Contact Sites Between the Endoplasmic Reticulum and Other Organelles. Annu Rev Cell Dev Biol.

[294] Luo J, Jiang LY, Yang H, Song BL (2019). Intracellular Cholesterol Transport by Sterol Transfer Proteins at Membrane Contact Sites. Trends Biochem Sci.

[295] Hanada K (2018). Lipid transfer proteins rectify inter-organelle flux and accurately deliver lipids at membrane contact sites. J Lipid Res.

[296] Worby CA, Dixon JE (2002). Sorting out the cellular functions of sorting nexins. Nat Rev Mol Cell Biol.

[297] Teasdale RD, Collins BM (2012). Insights into the PX (phox-homology) domain and SNX (sorting nexin) protein families: structures, functions and roles in disease. Biochem J.

[298] Teasdale RD, Loci D, Houghton F, Karlsson L, Gleeson PA (2001). A large family of endosome-localized proteins related to sorting nexin 1. Biochem J.

[299] Mas C, Norwood SJ, Bugarcic A, Kinna G, Leneva N, Kovtun O, Ghai R, Ona YL (2014). Structural basis for different phosphoinositide specificities of the PX domains of sorting nexins regulating G-protein signaling. J Biol Chem.

[300] Carroll P, Renoncourt Y, Gayet O, De Bovis B, Alonso S (2001). Sorting nexin-14, a gene expressed in motoneurons trapped by an in vitro preselection method. Dev Dyn.

[301] Datta S, Liu Y, Hariri H, Bowerman J, Henne WM (2019). Cerebellar ataxia disease-associated Snx14 promotes lipid droplet growth at ER-droplet contacts. J Cell Biol.

[302] Fenn J, Boursnell M, Hitti RJ, Jenkins CA, Terry RL, Priestnall SL, Kenny PJ, Mellersh CS (2016). Genome sequencing reveals a splice donor site mutation in the SNX14 gene associated with a novel cerebellar cortical degeneration in the Hungarian Vizsla dog breed. Bmc Genet.

[303] Thomas AC, Williams H, Seto-Salvia N, Bacchelli C, Jenkins D, O'Sullivan M, Mengrelis K, Ishida M (2014). Mutations in SNX14 cause a distinctive autosomal-recessive cerebellar ataxia and intellectual disability syndrome. Am J Hum Genet.

[304] Jazayeri R, Hu H, Fattahi Z, Musante L, Abedini SS, Hosseini M, Wienker TF, Ropers HH et al (2015) Exome Sequencing and Linkage Analysis Identified Novel Candidate Genes in Recessive Intellectual Disability Associated with Ataxia. Arch Iran Med 18(10):670-682. 0151810/AIM.007 26443249

[305] Shukla A, Upadhyai P, Shah J, Neethukrishna K, Bielas S, Girisha KM (2017). Autosomal recessive spinocerebellar ataxia 20: Report of a new patient and review of literature. Eur J Med Genet.

[306] Bryant D, Liu Y, Datta S, Hariri H, Seda M, Anderson G, Peskett E, Demetriou C (2018). SNX14 mutations affect endoplasmic reticulum-associated neutral lipid metabolism in autosomal recessive spinocerebellar ataxia 20. Hum Mol Genet.

[307] Akizu N, Cantagrel V, Zaki MS, Al-Gazali L, Wang X, Rosti RO, Dikoglu E, Gelot AB (2015). Biallelic mutations in SNX14 cause a syndromic form of cerebellar atrophy and lysosome-autophagosome dysfunction. Nat Genet.

[308] Henne WM, Zhu L, Balogi Z, Stefan C, Pleiss JA, Emr SD (2015). Mdm1/Snx13 is a novel ER-endolysosomal interorganelle tethering protein. J Cell Biol.

[309] Ebrahimi-Fakhari D (2018). Congenital Disorders of Autophagy: What a Pediatric Neurologist Should Know. Neuropediatrics.

[310] Hariri H, Rogers S, Ugrankar R, Liu YL, Feathers JR, Henne WM (2018). Lipid droplet biogenesis is spatially coordinated at ER-vacuole contacts under nutritional stress. Embo Rep.

[311] Hariri H, Speer N, Bowerman J, Rogers S, Fu G, Reetz E, Datta S, Feathers JR (2019). Mdm1 maintains endoplasmic reticulum homeostasis by spatially regulating lipid droplet biogenesis. J Cell Biol.

[312] Poppelreuther M, Rudolph B, Du C, Grossmann R, Becker M, Thiele C, Ehehalt R, Fullekrug J (2012). The N-terminal region of acyl-CoA synthetase 3 is essential for both the localization on lipid droplets and the function in fatty acid uptake. J Lipid Res.

[313] Poppelreuther M, Sander S, Minden F, Dietz MS, Exner T, Du C, Zhang I, Ehehalt F (1863). (2018) The metabolic capacity of lipid droplet localized acyl-CoA synthetase 3 is not sufficient to support local triglyceride synthesis independent of the endoplasmic reticulum in A431 cells. Biochim Biophys Acta Mol Cell Biol Lipids.

[314] Faergeman NJ, Black PN, Zhao XD, Knudsen J, Dirusso CC (2001). The Acyl-CoA synthetases encoded within FAA1 and FAA4 in Saccharomyces cerevisiae function as components of the fatty acid transport system linking import, activation, and intracellular Utilization. J Biol Chem.

[315] Fujimoto Y, Itabe H, Kinoshita T, Homma KJ, Onoduka J, Mori M, Yamaguchi S, Makita M (2007). Involvement of ACSL in local synthesis of neutral lipids in cytoplasmic lipid droplets in human hepatocyte HuH7. J Lipid Res.

[316] Yao H, Ye J (2008). Long chain acyl-CoA synthetase 3-mediated phosphatidylcholine synthesis is required for assembly of very low density lipoproteins in human hepatoma Huh7 cells. J Biol Chem.

[317] Nguyen TB, Olzmann JA (2019). Getting a handle on lipid droplets: Insights into ER-lipid droplet tethering. J Cell Biol.

[318] Engin AB (2017). What Is Lipotoxicity?. Adv Exp Med Biol.

[319] Feng B, Yao PM, Li Y, Devlin CM, Zhang D, Harding HP, Sweeney M, Rong JX (2003). The endoplasmic reticulum is the site of cholesterol-induced cytotoxicity in macrophages. Nat Cell Biol.

[320] Zoula S, Rijken PF, Peters JP, Farion R, Van der Sanden BP, Van der Kogel AJ, Decorps M, Remy C (2003). Pimonidazole binding in C6 rat brain glioma: relation with lipid droplet detection. Br J Cancer.

[321] Pennetta G, Welte MA (2018). Emerging Links between Lipid Droplets and Motor Neuron Diseases. Dev Cell.

[322] Rickman OJ, Baple EL, Crosby AH (2020). Lipid metabolic pathways converge in motor neuron degenerative diseases. Brain.

[323] Chaves-Filho AB, Pinto I, Dantas LS, Xavier AM, Inague A, Faria RL, Medeiros M, Glezer I (2019). Alterations in lipid metabolism of spinal cord linked to amyotrophic lateral sclerosis. Sci Rep.

[324] Gomez-Ramos P, Asuncion MM (2007). Ultrastructural localization of intraneuronal Abeta-peptide in Alzheimer disease brains. J Alzheimers Dis.

[325] Cole NB, Murphy DD, Grider T, Rueter S, Brasaemle D, Nussbaum RL (2002). Lipid droplet binding and oligomerization properties of the Parkinson's disease protein alpha-synuclein. J Biol Chem.

[326] Ouahoud S, Fiet MD, Martinez-Montanes F, Ejsing CS, Kuss O, Roden M, Markgraf DF (2018) Lipid droplet consumption is functionally coupled to vacuole homeostasis independent of lipophagy. J Cell Sci 131(11). 10.1242/jcs.213876 10.1242/jcs.213876 29678904

[327] Singh R, Kaushik S, Wang Y, Xiang Y, Novak I, Komatsu M, Tanaka K, Cuervo AM (2009). Autophagy regulates lipid metabolism. Nature.

[328] Watari H, Blanchette-Mackie EJ, Dwyer NK, Glick JM, Patel S, Neufeld EB, Brady RO, Pentchev PG (1999). Niemann-Pick C1 protein: obligatory roles for N-terminal domains and lysosomal targeting in cholesterol mobilization. Proc Natl Acad Sci U S A.

[329] Ioannou YA (2000). The structure and function of the Niemann-Pick C1 protein. Mol Genet Metab.

[330] Yu XH, Jiang N, Yao PB, Zheng XL, Cayabyab FS, Tang CK (2014). NPC1, intracellular cholesterol trafficking and atherosclerosis. Clin Chim Acta.

[331] Urano Y, Watanabe H, Murphy SR, Shibuya Y, Geng Y, Peden AA, Chang CC, Chang TY (2008). Transport of LDL-derived cholesterol from the NPC1 compartment to the ER involves the trans-Golgi network and the SNARE protein complex. Proc Natl Acad Sci U S A.

[332] Vanier MT (2010). Niemann-Pick disease type C. Orphanet J Rare Dis.

[333] Karten B, Peake KB, Vance JE (2009). Mechanisms and consequences of impaired lipid trafficking in Niemann-Pick type C1-deficient mammalian cells. Biochim Biophys Acta.

[334] Walkley SU, Suzuki K (2004). Consequences of NPC1 and NPC2 loss of function in mammalian neurons. Biochim Biophys Acta.

[335] Patterson MC, Hendriksz CJ, Walterfang M, Sedel F, Vanier MT, Wijburg F (2012). Recommendations for the diagnosis and management of Niemann-Pick disease type C: an update. Mol Genet Metab.

[336] Wang K, Xu R, Schrandt J, Shah P, Gong YZ, Preston C, Wang L, Yi JK (2015). Alkaline Ceramidase 3 Deficiency Results in Purkinje Cell Degeneration and Cerebellar Ataxia Due to Dyshomeostasis of Sphingolipids in the Brain. Plos Genet.

[337] Elrick MJ, Pacheco CD, Yu T, Dadgar N, Shakkottai VG, Ware C, Paulson HL, Lieberman AP (2010). Conditional Niemann-Pick C mice demonstrate cell autonomous Purkinje cell neurodegeneration. Hum Mol Genet.

[338] Yu T, Shakkottai VG, Chung C, Lieberman AP (2011). Temporal and cell-specific deletion establishes that neuronal Npc1 deficiency is sufficient to mediate neurodegeneration. Hum Mol Genet.

[339] Sarna JR, Larouche M, Marzban H, Sillitoe RV, Rancourt DE, Hawkes R (2003). Patterned Purkinje cell degeneration in mouse models of Niemann-Pick type C disease. J Comp Neurol.

[340] Yang F, Feng X, Rolfs A, Luo J (2018). Lovastatin promotes myelin formation in NPC1 mutant oligodendrocytes. J Neurol Sci.

[341] Yu T, Lieberman AP (2013). Npc1 acting in neurons and glia is essential for the formation and maintenance of CNS myelin. Plos Genet.

[342] Buard I, Pfrieger FW (2014). Relevance of neuronal and glial NPC1 for synaptic input to cerebellar Purkinje cells. Mol Cell Neurosci.

[343] Rabenstein M, Murr N, Hermann A, Rolfs A, Frech MJ (2019) Alteration of GABAergic Input Precedes Neurodegeneration of Cerebellar Purkinje Cells of NPC1-Deficient Mice. Int J Mol Sci 20(24). 10.3390/ijms20246288 10.3390/ijms20246288 PMC6940741 31847086

[344] Turley SD, Burns DK, Rosenfeld CR, Dietschy JM (1996). Brain does not utilize low density lipoprotein-cholesterol during fetal and neonatal development in the sheep. J Lipid Res.

[345] Quan G, Xie C, Dietschy JM, Turley SD (2003). Ontogenesis and regulation of cholesterol metabolism in the central nervous system of the mouse. Brain Res Dev Brain Res.

[346] Dietschy JM, Turley SD (2004). Thematic review series: brain Lipids. Cholesterol metabolism in the central nervous system during early development and in the mature animal. J Lipid Res.

[347] Mahley RW (2016). Central Nervous System Lipoproteins: ApoE and Regulation of Cholesterol Metabolism. Arterioscler Thromb Vasc Biol.

[348] Pitas RE, Boyles JK, Lee SH, Foss D, Mahley RW (1987). Astrocytes synthesize apolipoprotein E and metabolize apolipoprotein E-containing lipoproteins. Biochim Biophys Acta.

[349] Han X (2004). The role of apolipoprotein E in lipid metabolism in the central nervous system. Cell Mol Life Sci.

[350] Holtzman DM, Herz J, Bu G (2012). Apolipoprotein E and apolipoprotein E receptors: normal biology and roles in Alzheimer disease. Cold Spring Harb Perspect Med.

[351] Wang H, Eckel RH (2014). What are lipoproteins doing in the brain?. Trends Endocrinol Metab.

[352] Loving BA, Bruce KD (2020). Lipid and Lipoprotein Metabolism in Microglia. Front Physiol.

[353] Vitali C, Wellington CL, Calabresi L (2014). HDL and cholesterol handling in the brain. Cardiovasc Res.

[354] Wang N, Yvan-Charvet L, Lutjohann D, Mulder M, Vanmierlo T, Kim TW, Tall AR (2008). ATP-binding cassette transporters G1 and G4 mediate cholesterol and desmosterol efflux to HDL and regulate sterol accumulation in the brain. Faseb J.

[355] Herz J, Bock HH (2002). Lipoprotein receptors in the nervous system. Annu Rev Biochem.

[356] Dietschy JM, Turley SD (2001). Cholesterol metabolism in the brain. Curr Opin Lipidol.

[357] Flowers SA, Rebeck GW (2020). APOE in the normal brain. Neurobiol Dis.

[358] Bu G (2009). Apolipoprotein E and its receptors in Alzheimer's disease: pathways, pathogenesis and therapy. Nat Rev Neurosci.

[359] Maxfield FR, Iaea DB, Pipalia NH (2016). Role of STARD4 and NPC1 in intracellular sterol transport. Biochem Cell Biol.

[360] Pfisterer SG, Peranen J, Ikonen E (2016). LDL-cholesterol transport to the endoplasmic reticulum: current concepts. Curr Opin Lipidol.

[361] Hoglinger D, Burgoyne T, Sanchez-Heras E, Hartwig P, Colaco A, Newton J, Futter CE, Spiegel S (2019). NPC1 regulates ER contacts with endocytic organelles to mediate cholesterol egress. Nat Commun.

[362] Lim CY, Davis OB, Shin HR, Zhang J, Berdan CA, Jiang X, Counihan JL, Ory DS (2019). ER-lysosome contacts enable cholesterol sensing by mTORC1 and drive aberrant growth signalling in Niemann-Pick type C. Nat Cell Biol.

[363] Russell DW, Halford RW, Ramirez DM, Shah R, Kotti T (2009). Cholesterol 24-hydroxylase: an enzyme of cholesterol turnover in the brain. Annu Rev Biochem.

[364] Lund EG, Guileyardo JM, Russell DW (1999). cDNA cloning of cholesterol 24-hydroxylase, a mediator of cholesterol homeostasis in the brain. Proc Natl Acad Sci U S A.

[365] Lund EG, Xie C, Kotti T, Turley SD, Dietschy JM, Russell DW (2003). Knockout of the cholesterol 24-hydroxylase gene in mice reveals a brain-specific mechanism of cholesterol turnover. J Biol Chem.

[366] Frolov A, Zielinski SE, Crowley JR, Dudley-Rucker N, Schaffer JE, Ory DS (2003). NPC1 and NPC2 regulate cellular cholesterol homeostasis through generation of low density lipoprotein cholesterol-derived oxysterols. J Biol Chem.

[367] Repa JJ, Mangelsdorf DJ (2002). The liver X receptor gene team: potential new players in atherosclerosis. Nat Med.

[368] Nicoli ER, Al EN, Cluzeau CV, Wassif CA, Gray J, Burkert KR, Smith DA, Morris L (2016). Defective Cytochrome P450-Catalysed Drug Metabolism in Niemann-Pick Type C Disease. PLoS ONE.

[369] Kang I, Lee BC, Lee JY, Kim JJ, Sung EA, Lee SE, Shin N, Choi SW (2018). Stem cell-secreted 14,15- epoxyeicosatrienoic acid rescues cholesterol homeostasis and autophagic flux in Niemann-Pick-type C disease. Exp Mol Med.

[370] Mitroi DN, Pereyra-Gomez G, Soto-Huelin B, Senovilla F, Kobayashi T, Esteban JA, Ledesma MD (2019). NPC1 enables cholesterol mobilization during long-term potentiation that can be restored in Niemann-Pick disease type C by CYP46A1 activation. Embo Rep.

[371] Nobrega C, Mendonca L, Marcelo A, Lamaziere A, Tome S, Despres G, Matos CA, Mechmet F (2019). Restoring brain cholesterol turnover improves autophagy and has therapeutic potential in mouse models of spinocerebellar ataxia. Acta Neuropathol.

[372] Pacheco CD, Lieberman AP (2008). The pathogenesis of Niemann-Pick type C disease: a role for autophagy?. Expert Rev Mol Med.

[373] Lloyd-Evans E, Morgan AJ, He X, Smith DA, Elliot-Smith E, Sillence DJ, Churchill GC, Schuchman EH (2008). Niemann-Pick disease type C1 is a sphingosine storage disease that causes deregulation of lysosomal calcium. Nat Med.

[374] Colaco A, Fernandez-Suarez ME, Shepherd D, Gal L, Bibi C, Chuartzman S, Diot A, Morten K et al (2020) Unbiased yeast screens identify cellular pathways affected in Niemann-Pick disease type C. Life Sci Alliance 3(7). 10.26508/lsa.201800253 10.26508/lsa.201800253 PMC7283134 32487688

[375] Wheeler S, Schmid R, Sillence DJ (2019) Lipid(-)Protein Interactions in Niemann(-)Pick Type C Disease: Insights from Molecular Modeling. Int J Mol Sci 20(3). 10.3390/ijms20030717 10.3390/ijms20030717 PMC6387118 30736449

[376] Velayos-Baeza A, Vettori A, Copley RR, Dobson-Stone C, Monaco AP (2004). Analysis of the human VPS13 gene family. Genomics.

[377] Park JS, Halegoua S, Kishida S, Neiman AM (2015). A conserved function in phosphatidylinositol metabolism for mammalian Vps13 family proteins. PLoS ONE.

[378] Bankaitis VA, Johnson LM, Emr SD (1986). Isolation of yeast mutants defective in protein targeting to the vacuole. Proc Natl Acad Sci U S A.

[379] Rzepnikowska W, Flis K, Munoz-Braceras S, Menezes R, Escalante R, Zoladek T (2017). Yeast and other lower eukaryotic organisms for studies of Vps13 proteins in health and disease. Traffic.

[380] Lang AB, John PA, Walter P, Kornmann B (2015). ER-mitochondrial junctions can be bypassed by dominant mutations in the endosomal protein Vps13. J Cell Biol.

[381] Rubio JP, Danek A, Stone C, Chalmers R, Wood N, Verellen C, Ferrer X, Malandrini A (1997). Chorea-acanthocytosis: genetic linkage to chromosome 9q21. Am J Hum Genet.

[382] Duplomb L, Duvet S, Picot D, Jego G, El CS, Marle N, Gigot N, Aral B (2014). Cohen syndrome is associated with major glycosylation defects. Hum Mol Genet.

[383] Lesage S, Drouet V, Majounie E, Deramecourt V, Jacoupy M, Nicolas A, Cormier-Dequaire F, Hassoun SM (2016). Loss of VPS13C Function in Autosomal-Recessive Parkinsonism Causes Mitochondrial Dysfunction and Increases PINK1/Parkin-Dependent Mitophagy. Am J Hum Genet.

[384] Seong E, Insolera R, Dulovic M, Kamsteeg EJ, Trinh J, Bruggemann N, Sandford E, Li S (2018). Mutations in VPS13D lead to a new recessive ataxia with spasticity and mitochondrial defects. Ann Neurol.

[385] Gauthier J, Meijer IA, Lessel D, Mencacci NE, Krainc D, Hempel M, Tsiakas K, Prokisch H (2018). Recessive mutations in VPS13D cause childhood onset movement disorders. Ann Neurol.

[386] Koh K, Ishiura H, Shimazaki H, Tsutsumiuchi M, Ichinose Y, Nan H, Hamada S, Ohtsuka T (2020). VPS13D-related disorders presenting as a pure and complicated form of hereditary spastic paraplegia. Mol Genet Genomic Med.

[387] Kumar N, Leonzino M, Hancock-Cerutti W, Horenkamp FA, Li P, Lees JA, Wheeler H, Reinisch KM (2018). VPS13A and VPS13C are lipid transport proteins differentially localized at ER contact sites. J Cell Biol.

[388] Bean B, Dziurdzik SK, Kolehmainen KL, Fowler C, Kwong WK, Grad LI, Davey M, Schluter C (2018). Competitive organelle-specific adaptors recruit Vps13 to membrane contact sites. J Cell Biol.

[389] Murphy SE (1861). Levine TP (2016) VAP, a Versatile Access Point for the Endoplasmic Reticulum: Review and analysis of FFAT-like motifs in the VAPome. Biochim Biophys Acta.

[390] Anding AL, Wang C, Chang TK, Sliter DA, Powers CM, Hofmann K, Youle RJ, Baehrecke EH (2018). Vps13D Encodes a Ubiquitin-Binding Protein that Is Required for the Regulation of Mitochondrial Size and Clearance. Curr Biol.

[391] Auburger G, Gispert S, Torres-Odio S, Jendrach M, Brehm N, Canet-Pons J, Key J, Sen NE (2019) SerThr-PhosphoProteome of Brain from Aged PINK1-KO+A53T-SNCA Mice Reveals pT1928-MAP1B and pS3781-ANK2 Deficits, as Hub between Autophagy and Synapse Changes. Int J Mol Sci 20(13) 10.3390/ijms20133284 10.3390/ijms20133284 PMC6651490 31277379

[392] Krahmer N, Najafi B, Schueder F, Quagliarini F, Steger M, Seitz S, Kasper R, Salinas F (2018). Organellar Proteomics and Phospho-Proteomics Reveal Subcellular Reorganization in Diet-Induced Hepatic Steatosis. Dev Cell.

[393] Wong YC, Ysselstein D, Krainc D (2018). Mitochondria-lysosome contacts regulate mitochondrial fission via RAB7 GTP hydrolysis. Nature.

[394] Torres S, Balboa E, Zanlungo S, Enrich C, Garcia-Ruiz C, Fernandez-Checa JC (2017). Lysosomal and Mitochondrial Liaisons in Niemann-Pick Disease. Front Physiol.

[395] Insolera R, Lőrincz P, Wishnie AJ, Juhász G, Collins CA (2020) Vps13D is required for mitochondrial fission and mitophagy triggered by fission defects in Drosophila neurons 10.1101/2020.01.21.914523

[396] Dziurdzik SK, Bean B, Davey M, Conibear E (2020). A VPS13D spastic ataxia mutation disrupts the conserved adaptor-binding site in yeast Vps13. Hum Mol Genet.

[397] Liu SC, Lane WS, Lienhard GE (2000). Cloning and preliminary characterization of a 105 kDa protein with an N-terminal kinase-like domain. Biochim Biophys Acta.

[398] Schmidt WM, Kraus C, Hoger H, Hochmeister S, Oberndorfer F, Branka M, Bingemann S, Lassmann H (2007). Mutation in the Scyl1 gene encoding amino-terminal kinase-like protein causes a recessive form of spinocerebellar neurodegeneration. Embo Rep.

[399] Schmidt WM, Rutledge SL, Schule R, Mayerhofer B, Zuchner S, Boltshauser E, Bittner RE (2015). Disruptive SCYL1 Mutations Underlie a Syndrome Characterized by Recurrent Episodes of Liver Failure, Peripheral Neuropathy, Cerebellar Atrophy, and Ataxia. Am J Hum Genet.

[400] Shohet A, Cohen L, Haguel D, Mozer Y, Shomron N, Tzur S, Bazak L, Basel SL (2019). Variant in SCYL1 gene causes aberrant splicing in a family with cerebellar ataxia, recurrent episodes of liver failure, and growth retardation. Eur J Hum Genet.

[401] Chafe SC, Mangroo D (2010). Scyl1 facilitates nuclear tRNA export in mammalian cells by acting at the nuclear pore complex. Mol Biol Cell.

[402] Burman JL, Bourbonniere L, Philie J, Stroh T, Dejgaard SY, Presley JF, Mcpherson PS (2008). Scyl1, mutated in a recessive form of spinocerebellar neurodegeneration, regulates COPI-mediated retrograde traffic. J Biol Chem.

[403] Hamlin JN, Schroeder LK, Fotouhi M, Dokainish H, Ioannou MS, Girard M, Summerfeldt N, Melancon P (2014). Scyl1 scaffolds class II Arfs to specific subcomplexes of coatomer through the gamma-COP appendage domain. J Cell Sci.

[404] Xu X, Kedlaya R, Higuchi H, Ikeda S, Justice MJ, Setaluri V, Ikeda A (2010). Mutation in archain 1, a subunit of COPI coatomer complex, causes diluted coat color and Purkinje cell degeneration. Plos Genet.

[405] Beller M, Sztalryd C, Southall N, Bell M, Jackle H, Auld DS, Oliver B (2008). COPI complex is a regulator of lipid homeostasis. Plos Biol.

[406] Takashima K, Saitoh A, Hirose S, Nakai W, Kondo Y, Takasu Y, Kakeya H, Shin HW (2011). GBF1-Arf-COPI-ArfGAP-mediated Golgi-to-ER transport involved in regulation of lipid homeostasis. Cell Struct Funct.

[407] Wilfling F, Thiam AR, Olarte MJ, Wang J, Beck R, Gould TJ, Allgeyer ES, Pincet F (2014). Arf1/COPI machinery acts directly on lipid droplets and enables their connection to the ER for protein targeting. Elife.

[408] Thiam AR, Antonny B, Wang J, Delacotte J, Wilfling F, Walther TC, Beck R, Rothman JE (2013). COPI buds 60-nm lipid droplets from reconstituted water-phospholipid-triacylglyceride interfaces, suggesting a tension clamp function. Proc Natl Acad Sci U S A.

[409] Li C, Luo X, Zhao S, Siu GK, Liang Y, Chan HC, Satoh A, Yu SS (2017). COPI-TRAPPII activates Rab18 and regulates its lipid droplet association. Embo J.

[410] Li C, Yu SS (2016). Rab proteins as regulators of lipid droplet formation and lipolysis. Cell Biol Int.

[411] Berridge MJ (1993). Inositol trisphosphate and calcium signalling. Nature.

[412] Mak DO, Foskett JK (2015). Inositol 1,4,5-trisphosphate receptors in the endoplasmic reticulum: A single-channel point of view. Cell Calcium.

[413] Lock JT, Alzayady KJ, Yule DI, Parker I (2018) All three IP3 receptor isoforms generate Ca(2+) puffs that display similar characteristics. Sci Signal 11(561). 10.1126/scisignal.aau0344 10.1126/scisignal.aau0344 PMC6402561 30563861

[414] Parys JB, De Smedt H (2012). Inositol 1,4,5-trisphosphate and its receptors. Adv Exp Med Biol.

[415] Prole DL, Taylor CW (2016). Inositol 1,4,5-trisphosphate receptors and their protein partners as signalling hubs. J Physiol.

[416] Maeda N, Niinobe M, Mikoshiba K (1990). A cerebellar Purkinje cell marker P400 protein is an inositol 1,4,5-trisphosphate (InsP3) receptor protein. Purification and characterization of InsP3 receptor complex. Embo J.

[417] Walton PD, Airey JA, Sutko JL, Beck CF, Mignery GA, Sudhof TC, Deerinck TJ, Ellisman MH (1991). Ryanodine and inositol trisphosphate receptors coexist in avian cerebellar Purkinje neurons. J Cell Biol.

[418] Furuichi T, Simon-Chazottes D, Fujino I, Yamada N, Hasegawa M, Miyawaki A, Yoshikawa S, Guenet JL (1993). Widespread expression of inositol 1,4,5-trisphosphate receptor type 1 gene (Insp3r1) in the mouse central nervous system. Recept Channels.

[419] Sharp AH, Nucifora FJ, Blondel O, Sheppard CA, Zhang C, Snyder SH, Russell JT, Ryugo DK (1999). Differential cellular expression of isoforms of inositol 1,4,5-triphosphate receptors in neurons and glia in brain. J Comp Neurol.

[420] van de Leemput J, Chandran J, Knight MA, Holtzclaw LA, Scholz S, Cookson MR, Houlden H, Gwinn-Hardy K (2007). Deletion at ITPR1 underlies ataxia in mice and spinocerebellar ataxia 15 in humans. Plos Genet.

[421] Sugawara T, Hisatsune C, Le TD, Hashikawa T, Hirono M, Hattori M, Nagao S, Mikoshiba K (2013). Type 1 inositol trisphosphate receptor regulates cerebellar circuits by maintaining the spine morphology of purkinje cells in adult mice. J Neurosci.

[422] Terry LE, Alzayady KJ, Furati E, Yule DI (2018). Inositol 1,4,5-trisphosphate Receptor Mutations associated with Human Disease. Messenger (Los Angel).

[423] Zambonin JL, Bellomo A, Ben-Pazi H, Everman DB, Frazer LM, Geraghty MT, Harper AD, Jones JR (2017). Spinocerebellar ataxia type 29 due to mutations in ITPR1: a case series and review of this emerging congenital ataxia. Orphanet J Rare Dis.

[424] Synofzik M, Helbig KL, Harmuth F, Deconinck T, Tanpaiboon P, Sun B, Guo W, Wang R (2018). De novo ITPR1 variants are a recurrent cause of early-onset ataxia, acting via loss of channel function. Eur J Hum Genet.

[425] Gerber S, Alzayady KJ, Burglen L, Bremond-Gignac D, Marchesin V, Roche O, Rio M, Funalot B (2016). Recessive and Dominant De Novo ITPR1 Mutations Cause Gillespie Syndrome. Am J Hum Genet.

[426] Dentici ML, Barresi S, Nardella M, Bellacchio E, Alfieri P, Bruselles A, Pantaleoni F, Danieli A (2017). Identification of novel and hotspot mutations in the channel domain of ITPR1 in two patients with Gillespie syndrome. Gene.

[427] Sasaki M, Ohba C, Iai M, Hirabayashi S, Osaka H, Hiraide T, Saitsu H, Matsumoto N (2015). Sporadic infantile-onset spinocerebellar ataxia caused by missense mutations of the inositol 1,4,5-triphosphate receptor type 1 gene. J Neurol.

[428] van Dijk T, Barth P, Reneman L, Appelhof B, Baas F, Poll-The BT (2017). A de novo missense mutation in the inositol 1,4,5-triphosphate receptor type 1 gene causing severe pontine and cerebellar hypoplasia: Expanding the phenotype of ITPR1-related spinocerebellar ataxia's. Am J Med Genet A.

[429] Das J, Lilleker J, Shereef H, Ealing J (2017). Missense mutation in the ITPR1 gene presenting with ataxic cerebral palsy: Description of an affected family and literature review. Neurol Neurochir Pol.

[430] Hisatsune C, Mikoshiba K (2017). IP3 receptor mutations and brain diseases in human and rodents. J Neurochem.

[431] Ando H, Hirose M, Mikoshiba K (2018). Aberrant IP3 receptor activities revealed by comprehensive analysis of pathological mutations causing spinocerebellar ataxia 29. Proc Natl Acad Sci U S A.

[432] Nagaraja GM, Kandpal RP (2004). Chromosome 13q12 encoded Rho GTPase activating protein suppresses growth of breast carcinoma cells, and yeast two-hybrid screen shows its interaction with several proteins. Biochem Biophys Res Commun.

[433] Ponting CP, Aravind L (1999). START: a lipid-binding domain in StAR, HD-ZIP and signalling proteins. Trends Biochem Sci.

[434] Soccio RE, Breslow JL (2003). StAR-related lipid transfer (START) proteins: mediators of intracellular lipid metabolism. J Biol Chem.

[435] Alpy F, Tomasetto C (2005). Give lipids a START: the StAR-related lipid transfer (START) domain in mammals. J Cell Sci.

[436] Ng DC, Chan SF, Kok KH, Yam JW, Ching YP, Ng IO, Jin DY (2006). Mitochondrial targeting of growth suppressor protein DLC2 through the START domain. Febs Lett.

[437] Hatch GM, Gu Y, Xu FY, Cizeau J, Neumann S, Park JS, Loewen S, Mowat MR (2008). StARD13(Dlc-2) RhoGap mediates ceramide activation of phosphatidylglycerolphosphate synthase and drug response in Chinese hamster ovary cells. Mol Biol Cell.

[438] Subramanian M, Jayakumar S, Richhariya S, Hasan G (2013). Loss of IP3 receptor function in neuropeptide secreting neurons leads to obesity in adult Drosophila. Bmc Neurosci.

[439] Feriod CN, Oliveira AG, Guerra MT, Nguyen L, Richards KM, Jurczak MJ, Ruan HB, Camporez JP (2017). Hepatic Inositol 1,4,5 Trisphosphate Receptor Type 1 Mediates Fatty Liver. Hepatol Commun.

[440] Wang J, Lee J, Liem D, Ping P (2017). HSPA5 Gene encoding Hsp70 chaperone BiP in the endoplasmic reticulum. Gene.

[441] Wang X, Wang QC, Sun Z, Li T, Yang K, An C, Guo C, Tang TS (2019). ER stress mediated degradation of diacylglycerol acyltransferase impairs mitochondrial functions in TMCO1 deficient cells. Biochem Biophys Res Commun.

[442] Wang QC, Zheng Q, Tan H, Zhang B, Li X, Yang Y, Yu J, Liu Y (2016). TMCO1 Is an ER Ca(2+) Load-Activated Ca(2+) Channel. Cell.

[443] Liu J, Tang TS, Tu H, Nelson O, Herndon E, Huynh DP, Pulst SM, Bezprozvanny I (2009). Deranged calcium signaling and neurodegeneration in spinocerebellar ataxia type 2. J Neurosci.

[444] Rodriguez LR, Calap-Quintana P, Lapena-Luzon T, Pallardo FV, Schneuwly S, Navarro JA, Gonzalez-Cabo P (2020). Oxidative stress modulates rearrangement of endoplasmic reticulum-mitochondria contacts and calcium dysregulation in a Friedreich's ataxia model. Redox Biol.

[445] Koeppen AH, Mazurkiewicz JE (2013). Friedreich ataxia: neuropathology revised. J Neuropathol Exp Neurol.

[446] Coppola G, Marmolino D, Lu D, Wang Q, Cnop M, Rai M, Acquaviva F, Cocozza S, Pandolfo M, Geschwind DH (2009). Functional genomic analysis of frataxin deficiency reveals tissue-specific alterations and identifies the PPARgamma pathway as a therapeutic target in Friedreich's ataxia. Hum Mol Genet.

[447] Rodriguez-Pascau L, Britti E, Calap-Quintana P, Dong YN, Vergara C, Delaspre F, Medina-Carbonero M, Tamarit J, Pallardo FV, Gonzalez-Cabo P, Ros J, Lynch DR, Martinell M, Pizcueta P (2021). PPAR gamma agonist leriglitazone improves frataxin-loss impairments in cellular and animal models of Friedreich Ataxia. Neurobiol Dis.

[448] Navarro JA, Ohmann E, Sanchez D, Botella JA, Liebisch G, Molto MD, Ganfornina MD, Schmitz G, Schneuwly S (2010). Altered lipid metabolism in a Drosophila model of Friedreich's ataxia. Hum Mol Genet.

[449] Tamarit J, Obis E, Ros J (2016). Oxidative stress and altered lipid metabolism in Friedreich ataxia. Free Radic Biol Med.

[450] Chen K, Lin G, Haelterman NA, Ho TS, Li T, Li Z, Duraine L, Graham BH, Jaiswal M, Yamamoto S, Rasband MN, Bellen HJ (2016) Loss of Frataxin induces iron toxicity, sphingolipid synthesis, and Pdk1/Mef2 activation, leading to neurodegeneration. Elife 5. 10.7554/eLife.16043 10.7554/eLife.16043 PMC4956409 27343351

[451] Lin H, Magrane J, Clark EM, Halawani SM, Warren N, Rattelle A, Lynch DR (2017). Early VGLUT1-specific parallel fiber synaptic deficits and dysregulated cerebellar circuit in the KIKO mouse model of Friedreich ataxia. Dis Model Mech.

[452] Kemp KC, Cook AJ, Redondo J, Kurian KM, Scolding NJ, Wilkins A (2016). Purkinje cell injury, structural plasticity and fusion in patients with Friedreich's ataxia. Acta Neuropathol Commun.

[453] Conrard L, Tyteca D (2019) Regulation of Membrane Calcium Transport Proteins by the Surrounding Lipid Environment. Biomolecules 9(10). 10.3390/biom9100513 10.3390/biom9100513 PMC6843150 31547139

[454] Murata T, Lin MI, Stan RV, Bauer PM, Yu J, Sessa WC (2007). Genetic evidence supporting caveolae microdomain regulation of calcium entry in endothelial cells. J Biol Chem.

[455] Gueguinou M, Gambade A, Felix R, Chantome A, Fourbon Y, Bougnoux P, Weber G, Potier-Cartereau M (1848). (2015) Lipid rafts, KCa/ClCa/Ca2+ channel complexes and EGFR signaling: Novel targets to reduce tumor development by lipids?. Biochim Biophys Acta.

[456] Sampieri A, Santoyo K, Asanov A, Vaca L (2018). Association of the IP3R to STIM1 provides a reduced intraluminal calcium microenvironment, resulting in enhanced store-operated calcium entry. Sci Rep.

[457] Pani B, Ong HL, Liu X, Rauser K, Ambudkar IS, Singh BB (2008). Lipid rafts determine clustering of STIM1 in endoplasmic reticulum-plasma membrane junctions and regulation of store-operated Ca2+ entry (SOCE). J Biol Chem.

[458] Orci L, Ravazzola M, Le Coadic M, Shen WW, Demaurex N, Cosson P (2009). From the Cover: STIM1-induced precortical and cortical subdomains of the endoplasmic reticulum. Proc Natl Acad Sci U S A.

[459] Pacheco J, Dominguez L, Bohorquez-Hernandez A, Asanov A, Vaca L (2016). A cholesterol-binding domain in STIM1 modulates STIM1-Orai1 physical and functional interactions. Sci Rep.

[460] Derler I, Jardin I, Stathopulos PB, Muik M, Fahrner M, Zayats V, Pandey SK, Poteser M (2016). Cholesterol modulates Orai1 channel function. Sci Signal.

[461] Lupu VD, Kaznacheyeva E, Krishna UM, Falck JR, Bezprozvanny I (1998). Functional coupling of phosphatidylinositol 4,5-bisphosphate to inositol 1,4,5-trisphosphate receptor. J Biol Chem.

[462] Shyu PJ, Ng B, Ho N, Chaw R, Seah YL, Marvalim C, Thibault G (2019). Membrane phospholipid alteration causes chronic ER stress through early degradation of homeostatic ER-resident proteins. Sci Rep.

[463] Wangeline MA, Vashistha N, Hampton RY (2017). Proteostatic Tactics in the Strategy of Sterol Regulation. Annu Rev Cell Dev Biol.

[464] Volmer R, Ron D (2015). Lipid-dependent regulation of the unfolded protein response. Curr Opin Cell Biol.

[465] Kitai Y, Ariyama H, Kono N, Oikawa D, Iwawaki T, Arai H (2013). Membrane lipid saturation activates IRE1alpha without inducing clustering. Genes Cells.

[466] Kono N, Amin-Wetzel N, Ron D (2017). Generic membrane-spanning features endow IRE1alpha with responsiveness to membrane aberrancy. Mol Biol Cell.

[467] Tam AB, Roberts LS, Chandra V, Rivera IG, Nomura DK, Forbes DJ, Niwa M (2018). The UPR Activator ATF6 Responds to Proteotoxic and Lipotoxic Stress by Distinct Mechanisms. Dev Cell.

[468] Klemm EJ, Spooner E, Ploegh HL (2011). Dual role of ancient ubiquitous protein 1 (AUP1) in lipid droplet accumulation and endoplasmic reticulum (ER) protein quality control. J Biol Chem.

[469] Spandl J, Lohmann D, Kuerschner L, Moessinger C, Thiele C (2011). Ancient ubiquitous protein 1 (AUP1) localizes to lipid droplets and binds the E2 ubiquitin conjugase G2 (Ube2g2) via its G2 binding region. J Biol Chem.

[470] Jo Y, Hartman IZ, Debose-Boyd RA (2013). Ancient ubiquitous protein-1 mediates sterol-induced ubiquitination of 3-hydroxy-3-methylglutaryl CoA reductase in lipid droplet-associated endoplasmic reticulum membranes. Mol Biol Cell.

[471] Wang CW, Lee SC (2012). The ubiquitin-like (UBX)-domain-containing protein Ubx2/Ubxd8 regulates lipid droplet homeostasis. J Cell Sci.

[472] Olzmann JA, Richter CM, Kopito RR (2013). Spatial regulation of UBXD8 and p97/VCP controls ATGL-mediated lipid droplet turnover. Proc Natl Acad Sci U S A.

[473] Ruggiano A, Mora G, Buxo L, Carvalho P (2016). Spatial control of lipid droplet proteins by the ERAD ubiquitin ligase Doa10. Embo J.

[474] Straiker A, Wager-Miller J, Hu SS, Blankman JL, Cravatt BF, Mackie K (2011). COX-2 and fatty acid amide hydrolase can regulate the time course of depolarization-induced suppression of excitation. Br J Pharmacol.

[475] Yuan C, Smith WL (2015). A cyclooxygenase-2-dependent prostaglandin E2 biosynthetic system in the Golgi apparatus. J Biol Chem.

[476] Lu JP, Wang Y, Sliter DA, Pearce MM, Wojcikiewicz RJ (2011). RNF170 protein, an endoplasmic reticulum membrane ubiquitin ligase, mediates inositol 1,4,5-trisphosphate receptor ubiquitination and degradation. J Biol Chem.

[477] Wright FA, Lu JP, Sliter DA, Dupre N, Rouleau GA, Wojcikiewicz RJ (2015). A Point Mutation in the Ubiquitin Ligase RNF170 That Causes Autosomal Dominant Sensory Ataxia Destabilizes the Protein and Impairs Inositol 1,4,5-Trisphosphate Receptor-mediated Ca2+ Signaling. J Biol Chem.

[478] Pearce MM, Wang Y, Kelley GG, Wojcikiewicz RJ (2007). SPFH2 mediates the endoplasmic reticulum-associated degradation of inositol 1,4,5-trisphosphate receptors and other substrates in mammalian cells. J Biol Chem.

[479] Wang Y, Pearce MM, Sliter DA, Olzmann JA, Christianson JC, Kopito RR, Boeckmann S, Gagen C (2009). SPFH1 and SPFH2 mediate the ubiquitination and degradation of inositol 1,4,5-trisphosphate receptors in muscarinic receptor-expressing HeLa cells. Biochim Biophys Acta.

[480] Pearce MM, Wormer DB, Wilkens S, Wojcikiewicz RJ (2009). An endoplasmic reticulum (ER) membrane complex composed of SPFH1 and SPFH2 mediates the ER-associated degradation of inositol 1,4,5-trisphosphate receptors. J Biol Chem.

[481] Yang B, Qu M, Wang R, Chatterton JE, Liu XB, Zhu B, Narisawa S, Millan JL et al (2015) The critical role of membralin in postnatal motor neuron survival and disease. Elife 4. 10.7554/eLife.06500 10.7554/eLife.06500 PMC4460860 25977983

[482] Sha H, Sun S, Francisco AB, Ehrhardt N, Xue Z, Liu L, Lawrence P, Mattijssen F (2014). The ER-associated degradation adaptor protein Sel1L regulates LPL secretion and lipid metabolism. Cell Metab.

[483] Kyostila K, Cizinauskas S, Seppala EH, Suhonen E, Jeserevics J, Sukura A, Syrja P, Lohi H (2012). A SEL1L mutation links a canine progressive early-onset cerebellar ataxia to the endoplasmic reticulum-associated protein degradation (ERAD) machinery. Plos Genet.

[484] Bublitz SK, Alhaddad B, Synofzik M, Kuhl V, Lindner A, Freiberg C, Schmidt H, Strom TM (2017). Expanding the phenotype of DNAJC3 mutations: A case with hypothyroidism additionally to diabetes mellitus and multisystemic neurodegeneration. Clin Genet.

[485] Anttonen AK, Mahjneh I, Hamalainen RH, Lagier-Tourenne C, Kopra O, Waris L, Anttonen M, Joensuu T (2005). The gene disrupted in Marinesco-Sjogren syndrome encodes SIL1, an HSPA5 cochaperone. Nat Genet.

[486] Senderek J, Krieger M, Stendel C, Bergmann C, Moser M, Breitbach-Faller N, Rudnik-Schoneborn S, Blaschek A (2005). Mutations in SIL1 cause Marinesco-Sjogren syndrome, a cerebellar ataxia with cataract and myopathy. Nat Genet.

[487] Synofzik M, Haack TB, Kopajtich R, Gorza M, Rapaport D, Greiner M, Schonfeld C, Freiberg C (2014). Absence of BiP co-chaperone DNAJC3 causes diabetes mellitus and multisystemic neurodegeneration. Am J Hum Genet.

[488] Kizhakkedath P, John A, Al-Gazali L, Ali BR (2018). Degradation routes of trafficking-defective VLDLR mutants associated with Dysequilibrium syndrome. Sci Rep.

[489] Chung KT, Shen Y, Hendershot LM (2002). BAP, a mammalian BiP-associated protein, is a nucleotide exchange factor that regulates the ATPase activity of BiP. J Biol Chem.

[490] Petrova K, Oyadomari S, Hendershot LM, Ron D (2008). Regulated association of misfolded endoplasmic reticulum lumenal proteins with P58/DNAJc3. Embo J.

[491] Rutkowski DT, Kang SW, Goodman AG, Garrison JL, Taunton J, Katze MG, Kaufman RJ, Hegde RS (2007). The role of p58IPK in protecting the stressed endoplasmic reticulum. Mol Biol Cell.

[492] Cook KL, Soto-Pantoja DR, Clarke PA, Cruz MI, Zwart A, Warri A, Hilakivi-Clarke L, Roberts DD (2016). Endoplasmic Reticulum Stress Protein GRP78 Modulates Lipid Metabolism to Control Drug Sensitivity and Antitumor Immunity in Breast Cancer. Cancer Res.

[493] Ye R, Jung DY, Jun JY, Li J, Luo S, Ko HJ, Kim JK, Lee AS (2010). Grp78 heterozygosity promotes adaptive unfolded protein response and attenuates diet-induced obesity and insulin resistance. Diabetes.

[494] Rutkowski DT, Wu J, Back SH, Callaghan MU, Ferris SP, Iqbal J, Clark R, Miao H (2008). UPR pathways combine to prevent hepatic steatosis caused by ER stress-mediated suppression of transcriptional master regulators. Dev Cell.

[495] Bertolotti A, Zhang Y, Hendershot LM, Harding HP, Ron D (2000). Dynamic interaction of BiP and ER stress transducers in the unfolded-protein response. Nat Cell Biol.

[496] Shen J, Snapp EL, Lippincott-Schwartz J, Prywes R (2005). Stable binding of ATF6 to BiP in the endoplasmic reticulum stress response. Mol Cell Biol.

[497] Kammoun HL, Chabanon H, Hainault I, Luquet S, Magnan C, Koike T, Ferre P, Foufelle F (2009). GRP78 expression inhibits insulin and ER stress-induced SREBP-1c activation and reduces hepatic steatosis in mice. J Clin Invest.

[498] Yan W, Frank CL, Korth MJ, Sopher BL, Novoa I, Ron D, Katze MG (2002). Control of PERK eIF2alpha kinase activity by the endoplasmic reticulum stress-induced molecular chaperone P58IPK. Proc Natl Acad Sci U S A.

[499] Bobrovnikova-Marjon E, Pytel D, Riese MJ, Vaites LP, Singh N, Koretzky GA, Witze ES, Diehl JA (2012). PERK utilizes intrinsic lipid kinase activity to generate phosphatidic acid, mediate Akt activation, and promote adipocyte differentiation. Mol Cell Biol.

[500] Bobrovnikova-Marjon E, Hatzivassiliou G, Grigoriadou C, Romero M, Cavener DR, Thompson CB, Diehl JA (2008). PERK-dependent regulation of lipogenesis during mouse mammary gland development and adipocyte differentiation. Proc Natl Acad Sci U S A.

