# Supplementary material for: Lipid Dyshomeostasis and Inherited Cerebellar Ataxia
Source: Mol Neurobiol. 2022 Apr 14;59(6):3800–28. doi: 10.1007/s12035-022-02826-2 (PMC9148275; doi:10.1007/s12035-022-02826-2)
Supplement: Supplementary file 1 — Supplementary file1 (DOCX 71 KB) [file 12035_2022_2826_MOESM1_ESM.docx]

**Table S1.** Summary of the numbers of patients with or without ataxia related to genes for lipid homeostasis

| **Gene** | **Type of Mutation** | **Present of Mutation** | **Ataxia** | **No Ataxia** | **Total** | **Ataxia** | **No Ataxia** | **Ref** |
| --- | --- | --- | --- | --- | --- | --- | --- | --- |
| **ABHD12** | p.Asp113PhefsX15 | + | 3 | 5 | 37 | 22 | 15 | Fiskerstrand et al. (2010) |
|  |  | - | 19 | 10 |  |  |  |  |
|  | del14007insGG | + | 3 | 0 |  |  |  |  |
|  |  | - | 19 | 15 |  |  |  |  |
|  | p.Arg352X | + | 1 | 0 |  |  |  |  |
|  |  | - | 21 | 15 |  |  |  |  |
|  | p.His285fsX1 | + | 6 | 1 |  |  |  |  |
|  |  | - | 16 | 14 |  |  |  |  |
|  | p.Arg262* | + | 2 | 0 |  |  |  | Thimm et al. (2020) |
|  |  | - | 20 | 15 |  |  |  |  |
|  | p.Arg65X | + | 1 | 1 |  |  |  | Eisenberger et al. (2012) |
|  |  | - | 21 | 14 |  |  |  |  |
|  | a novel splice site mutation (c.316+2T>A) in intron 2 | + | 1 | 1 |  |  |  | Yoshimura et al. (2015) |
|  |  | - | 21 | 14 |  |  |  |  |
|  | p.Tyr83X | + | 0 | 2 |  |  |  | Li T et al (2019) |
|  |  | - | 22 | 13 |  |  |  |  |
|  | p.Arg71Tyrfs*26 | + | 1 | 1 |  |  |  | Frasquet et al (2018) |
|  |  | - | 21 | 14 |  |  |  |  |
|  | p.Asn127Aspfs*23 | + | 1 | 0 |  |  |  | Lerat et al (2017) |
|  |  | - | 21 | 15 |  |  |  |  |
|  | p.T253R | + | 1 | 0 |  |  |  | Tingaud-Sequeira et al (2017) |
|  |  | - | 21 | 15 |  |  |  |  |
|  | p.Arg107Glufs*8；p.Thr202Ile | + | 0 | 4 |  |  |  | Nishiguchi et al (2014) |
|  |  | - | 22 | 11 |  |  |  |  |
|  | p.His372Gln | + | 1 | 0 |  |  |  |  |
|  |  | - | 21 | 15 |  |  |  |  |
|  | p.Trp159*; p.Arg186Pro | + | 1 | 0 |  |  |  |  |
|  |  | - | 21 | 15 |  |  |  |  |
| **BAP** | 1029+1G→A,skipping exon9(hom) | + | 1 | 0 | 40 | 38 | 2 | Senderek et al. (2005) |
|  |  | - | 37 | 2 |  |  |  |  |
|  | 645+1G→A,skipping exon6(hom) | + | 1 | 0 |  |  |  |  |
|  |  | - | 37 | 2 |  |  |  |  |
|  | p.R111X | + | 7 | 0 |  |  |  | Senderek et al. (2005)、 Anttonen et al. (2005) |
|  |  | - | 31 | 2 |  |  |  |  |
|  | p.L316fs (het);  p.M344fs (het) | + | 2 | 0 |  |  |  | Senderek et al. (2005) |
|  |  | - | 36 | 2 |  |  |  |  |
|  | p.L316fs (het);  p.L456fs (het) | + | 1 | 0 |  |  |  |  |
|  |  | - | 37 | 2 |  |  |  |  |
|  | p. E60X (het);  p.G116fs (het) | + | 2 | 0 |  |  |  |  |
|  |  | - | 36 | 2 |  |  |  |  |
|  | p.Q417X (hom) | + | 1 | 0 |  |  |  |  |
|  |  | - | 37 | 2 |  |  |  |  |
|  | p.D170EfsX4 | + | 5 | 0 |  |  |  | Anttonen et al. (2005) |
|  |  | - | 33 | 2 |  |  |  |  |
|  | p.D170EfsX4;  p.V186_Q215del;  p.A152_Q215del | + | 2 | 0 |  |  |  |  |
|  |  | - | 36 | 2 |  |  |  |  |
|  | p.H71QfsX5 | + | 1 | 0 |  |  |  |  |
|  |  | - | 37 | 2 |  |  |  |  |
|  | p.Q438X | + | 2 | 2 |  |  |  | Karim et al. (2006) |
|  |  | - | 36 | 0 |  |  |  |  |
|  | p.L457P | + | 2 | 0 |  |  |  | anttomen et al 2008 |
|  |  | - | 36 | 2 |  |  |  |  |
|  | p.Leu313AlafsTer39 | + | 3 | 0 |  |  |  |  |
|  |  | - | 35 | 2 |  |  |  |  |
|  | c.1030-9G＞A | + | 4 | 0 |  |  |  |  |
|  |  | - | 34 | 2 |  |  |  |  |
|  | c.1367T＞A | + | 1 | 0 |  |  |  |  |
|  |  | - | 37 | 2 |  |  |  |  |
|  | 598delGAAGA；58-kb deletion in exon 6 | + | 3 | 0 |  |  |  | Takahata et al 2010 |
|  |  | - | 35 | 2 |  |  |  |  |
| DNAJC3 | p.R194X | + | 4 | 0 | 8 | 8 | 0 | Bublitz et al.(2017) |
|  |  | - | 4 | 0 |  |  |  |  |
|  | Deletion of exons 6-12 | + | 2 | 0 |  |  |  | Synofzik (2015) |
|  |  | - | 6 | 0 |  |  |  |  |
|  | c.393+2T＞G | + | 2 | 0 |  |  |  | Z Alev Ozon et al.(2020) |
|  |  | - | 6 | 0 |  |  |  |  |
| ELOVL4 | p.L168F | + | 21 | 7 | 77 | 34 | 43 | Cadieux-Dion et al.(2014) |
|  |  | - | 13 | 36 |  |  |  |  |
|  | p.W246G | + | 9 | 0 |  |  |  | Ozaki et al. (2015) |
|  |  | - | 25 | 43 |  |  |  |  |
|  | p.T233M | + | 3 | 0 |  |  |  | Ozaki et al. (2019) |
|  |  | - | 31 | 43 |  |  |  |  |
|  | p.Q180P | + | 1 | 0 |  |  |  | Bourassa et al.2015 |
|  |  | - | 33 | 43 |  |  |  |  |
|  | 797–801delAACT | + | 0 | 15 |  |  |  | Zhang et al 2001 |
|  |  | - | 34 | 28 |  |  |  |  |
|  | 2-bp del, 790T and 794T | + | 0 | 15 |  |  |  | Bernstein et al 2001 |
|  |  | - | 34 | 28 |  |  |  |  |
|  | p.Tyr270X | + | 0 | 4 |  |  |  | Maugeri et al 2004 |
|  |  | - | 34 | 39 |  |  |  |  |
|  | p.R216X | + | 0 | 2 |  |  |  | Aldahmesh et al 2011 |
|  |  | - | 34 | 41 |  |  |  |  |
| ELOVL5 | p.Gly230Val | + | 21 | 0 | 28 | 28 | 0 | Di Gregorio et al. (2014) |
|  |  | - | 7 | 0 |  |  |  |  |
|  | p.Leu72Val | + | 2 | 0 |  |  |  |  |
|  |  | - | 26 | 0 |  |  |  |  |
|  | p.Tyr260Cys | + | 5 | 0 |  |  |  | Gazulla J et al. 2020 |
|  |  | - | 23 | 0 |  |  |  |  |
| FA2H | c.786þ1G/A mutation | + | 7 | 0 | 26 | 26 | 0 | Edvardson et al. (2008) |
|  |  | - | 19 | 0 |  |  |  |  |
|  | p.D35Y | + | 2 | 0 |  |  |  |  |
|  |  | - | 24 | 0 |  |  |  |  |
|  | p.Arg235Cys | + | 7 | 0 |  |  |  | Dick et al. (2010) |
|  |  | - | 19 | 0 |  |  |  |  |
|  | p.Arg53_Ile58del | + | 2 | 0 |  |  |  |  |
|  |  | - | 24 | 0 |  |  |  |  |
|  | p.R154C | + | 3 | 0 |  |  |  | Kruer et al 2010 |
|  |  | - | 23 | 0 |  |  |  |  |
|  | p.Y170X | + | 4 | 0 |  |  |  |  |
|  |  | - | 22 | 0 |  |  |  |  |
|  | p.F236S | + | 1 | 0 |  |  |  | Pierson et al. (2012) |
|  |  | - | 25 | 0 |  |  |  |  |
| GBA2 | p. Arg630Trp | + | 6 | 0 | 29 | 25 | 4 | Martin et al. (2013) |
|  |  | - | 19 | 4 |  |  |  |  |
|  | p.Arg234* | + | 2 | 0 |  |  |  |  |
|  |  | - | 23 | 4 |  |  |  |  |
|  | p.W173X； p.Thr492Argfs*9 | + | 3 | 0 |  |  |  |  |
|  |  | - | 22 | 4 |  |  |  |  |
|  | p.Tyr121* | + | 3 | 0 |  |  |  | Hammer et al. (2013) |
|  |  | - | 22 | 4 |  |  |  |  |
|  | p.Arg340* | + | 5 | 0 |  |  |  |  |
|  |  | - | 20 | 4 |  |  |  |  |
|  | p.Arg873His | + | 1 | 1 |  |  |  |  |
|  |  | - | 24 | 3 |  |  |  |  |
|  | in the splice acceptor site of exon 3 causing a complete loss of GBA2 mRNA expression | + | 1 | 0 |  |  |  | Coarelli et al 2018 |
|  |  | - | 24 | 4 |  |  |  |  |
|  | p. Cys688Tyr | + | 0 | 1 |  |  |  | Spagnoli et al 2020 |
|  |  | - | 25 | 3 |  |  |  |  |
|  | p.Gly683Arg | + | 1 | 2 |  |  |  | Citterio et al 2014 |
|  |  | - | 24 | 2 |  |  |  |  |
|  | p.Met510-Valfs*17 | + | 3 | 0 |  |  |  | Haugarvoll et al 2017 |
|  |  | - | 22 | 4 |  |  |  |  |
| ITPR1 | a heterozygous deletion of exons 1 to 48 | + | 14 | 0 | 69 | 69 | 0 | Iwaki et al 2008 |
|  |  | - | 55 | 0 |  |  |  |  |
|  | p.P1059L | + | 11 | 0 |  |  |  | Hara et al. (2008) |
|  |  | - | 58 | 0 |  |  |  |  |
|  | p.V1553M | + | 24 | 0 |  |  |  | Huang et al. (2012) |
|  |  | - | 45 | 0 |  |  |  |  |
|  | p.N602D | + | 1 | 0 |  |  |  | Parolin Schnekenberg et al. (2015) |
|  |  | - | 68 | 0 |  |  |  |  |
|  | p.Q1558X | + | 1 | 0 |  |  |  | Gerber et al. (2016) |
|  |  | - | 68 | 0 |  |  |  |  |
|  | p.R728X | + | 1 | 0 |  |  |  |  |
|  |  | - | 68 | 0 |  |  |  |  |
|  | p.Gly2102Valfs5Ter;  p.Ala2221Valfs23Ter | + | 1 | 0 |  |  |  |  |
|  |  | - | 68 | 0 |  |  |  |  |
|  | p.K2563del | + | 1 | 0 |  |  |  |  |
|  |  | - | 68 | 0 |  |  |  |  |
|  | p.F2553L | + | 1 | 0 |  |  |  |  |
|  |  | - | 68 | 0 |  |  |  |  |
|  | p.K2596del | + | 4 | 0 |  |  |  | McEntagart et al. (2016) |
|  |  | - | 65 | 0 |  |  |  |  |
|  | p.E2094N | + | 1 | 0 |  |  |  |  |
|  |  | - | 68 | 0 |  |  |  |  |
|  | p.E2094G | + | 2 | 0 |  |  |  |  |
|  |  | - | 67 | 0 |  |  |  |  |
|  | p.G2539R;  c.7615G-A | + | 5 | 0 |  |  |  |  |
|  |  | - | 64 | 0 |  |  |  |  |
|  | p.G2539R;  c.7615G-C | + | 1 | 0 |  |  |  |  |
|  |  | - | 68 | 0 |  |  |  |  |
|  | p.I2550N | + | 1 | 0 |  |  |  | van Dijk et al. (2017) |
|  |  | - | 68 | 0 |  |  |  |  |
| PNPLA6 | p.M1012V | + | 3 | 0 | 33 | 26 | 7 | Rainier et al. (2008) |
|  |  | - | 23 | 7 |  |  |  |  |
|  | p.R890H;  p.Ser982fs1019 | + | 2 | 0 |  |  |  |  |
|  |  | - | 24 | 7 |  |  |  |  |
|  | p.T1058I | + | 4 | 0 |  |  |  | Synofzik et al. (2014) |
|  |  | - | 22 | 7 |  |  |  |  |
|  | p:Val738GlnfsTer98;  p.V1110M | + | 2 | 0 |  |  |  |  |
|  |  | - | 24 | 7 |  |  |  |  |
|  | p.G578W;  p.F1066S | + | 2 | 0 |  |  |  |  |
|  |  | - | 24 | 7 |  |  |  |  |
|  | p.V263I;  p.G840E | + | 1 | 0 |  |  |  |  |
|  |  | - | 25 | 7 |  |  |  |  |
|  | p.Arg1031Glufs*38;p.Arg1362Gly | + | 1 | 0 |  |  |  |  |
|  |  | - | 25 | 7 |  |  |  |  |
|  | p.Val263Ile;  p.Gly840Glu | + | 0 | 1 |  |  |  |  |
|  |  | - | 26 | 6 |  |  |  |  |
|  | p.Arg1031Glufs*38;p.Val1100Gly | + | 1 | 0 |  |  |  |  |
|  |  | - | 25 | 7 |  |  |  |  |
|  | p.Asp376GlyfsTer18;  p.R1099C | + | 2 | 0 |  |  |  | Topaloglu et al. (2014) |
|  |  | - | 24 | 7 |  |  |  |  |
|  | p.S1127C | + | 2 | 0 |  |  |  |  |
|  |  | - | 24 | 7 |  |  |  |  |
|  | p.R1311W;  p.G832fsX13 | + | 1 | 1 |  |  |  |  |
|  |  | - | 25 | 6 |  |  |  |  |
|  | p.Arg1031fs*38;  p.G1129R | + | 1 | 1 |  |  |  | Hufnagel et al. (2015) |
|  |  | - | 25 | 6 |  |  |  |  |
|  | p.Arg1031fs*38 ;  p.G726R | + | 4 | 0 |  |  |  |  |
|  |  | - | 22 | 7 |  |  |  |  |
|  | p.G1176S;  p.R1099Q | + | 0 | 2 |  |  |  |  |
|  |  | - | 26 | 5 |  |  |  |  |
|  | c.1973+2T>G;  p.Val1215Ala | + | 0 | 1 |  |  |  |  |
|  |  | - | 26 | 6 |  |  |  |  |
|  | dup(Ex14–20);  p.Val1215Ala | + | 0 | 1 |  |  |  |  |
|  |  | - | 26 | 6 |  |  |  |  |
| Rubicon | p.Ala875ValfsTer146 | + | 5 | 0 | 5 | 5 | 0 | Assoum et al. (2013)、Seidahmed et al. (2020) |
|  |  | - | 0 | 0 |  |  |  |  |
| SCYL1 | p.Val313CysfsTer6 p.Ala504ProfsTer15 | + | 2 | 0 | 11 | 4 | 7 | Schmidt et al. (2015) |
|  |  | - | 2 | 7 |  |  |  |  |
|  | loss of residues 373-410;  p.Q546X | + | 1 | 0 |  |  |  |  |
|  |  | - | 3 | 7 |  |  |  |  |
|  | p.Q628X | + | 0 | 1 |  |  |  | Lenz et al. (2018) |
|  |  | - | 4 | 6 |  |  |  |  |
|  | p.D478G | + | 0 | 2 |  |  |  |  |
|  |  | - | 4 | 5 |  |  |  |  |
|  | p.E86X | + | 0 | 1 |  |  |  |  |
|  |  | - | 4 | 6 |  |  |  |  |
|  | p.Q57X | + | 0 | 2 |  |  |  |  |
|  |  | - | 4 | 5 |  |  |  |  |
|  | p.A105V | + | 0 | 1 |  |  |  |  |
|  |  | - | 4 | 6 |  |  |  |  |
|  | p.Cys512LeufsTer8 | + | 1 | 0 |  |  |  | Spagnoli et al. (2019) |
|  |  | - | 3 | 7 |  |  |  |  |
| SEL1L | p.Ser658Pro(dog) | + | 11 | 0 | 11 | 11 | 0 | Kyöstilä K et al. (2014) |
|  |  | - | 0 | 0 |  |  |  |  |
| SNX14 | P.Q866X | + | 2 | 0 | 29 | 29 | 0 | Thomas et al. (2014) |
|  |  | - | 27 | 0 |  |  |  |  |
|  | p.Val369_Leu702del | + | 4 | 0 |  |  |  |  |
|  |  | - | 25 | 0 |  |  |  |  |
|  | p.Ala603_Gly632del | + | 1 | 0 |  |  |  |  |
|  |  | - | 28 | 0 |  |  |  |  |
|  | p.R378X | + | 15 | 0 |  |  |  | Akizu et al. (2015) |
|  |  | - | 14 | 0 |  |  |  |  |
|  | p.Glu216ArgfsTer25 | + | 3 | 0 |  |  |  |  |
|  |  | - | 26 | 0 |  |  |  |  |
|  | p.L143X | + | 1 | 0 |  |  |  |  |
|  |  | - | 28 | 0 |  |  |  |  |
|  | p.Cys890Ter | + | 3 | 0 |  |  |  |  |
|  |  | - | 26 | 0 |  |  |  |  |
| SRD5A3 | 286delCAAinsTGAGTAAGGC | + | 4 | 2 | 14 | 8 | 6 | Cantagrel et al. (2010) |
|  |  | - | 4 | 4 |  |  |  |  |
|  | p.Leu98ValfsX121 | + | 1 | 0 |  |  |  |  |
|  |  | - | 7 | 6 |  |  |  |  |
|  | p.W107X | + | 1 | 0 |  |  |  |  |
|  |  | - | 7 | 6 |  |  |  |  |
|  | p.R142X;  p.Y163X | + | 1 | 0 |  |  |  |  |
|  |  | - | 7 | 6 |  |  |  |  |
|  | p.S10X | + | 0 | 1 |  |  |  |  |
|  |  | - | 8 | 5 |  |  |  |  |
|  | Genomic rearrangement | + | 1 | 0 |  |  |  |  |
|  |  | - | 7 | 6 |  |  |  |  |
|  | 203dupC | + | 0 | 3 |  |  |  | Kahrizi et al. (2011) |
|  |  | - | 8 | 3 |  |  |  |  |
| TMEM16K | p.L510R | + | 3 | 0 | 14 | 14 | 0 | Vermeer et al. (2010) |
|  |  | - | 11 | 0 |  |  |  |  |
|  | p.Leu384fs | + | 6 | 0 |  |  |  | Vermeer et al. (2011)、Balreira et al. (2014) |
|  |  | - | 8 | 0 |  |  |  |  |
|  | 1476+1G＞T;p.Leu535* | + | 2 | 0 |  |  |  | Vermeer et al. (2012) |
|  |  | - | 12 | 0 |  |  |  |  |
|  | p.Tyr203* | + | 1 | 0 |  |  |  | Balreira et al. (2014) |
|  |  | - | 13 | 0 |  |  |  |  |
|  | p.Asp45Argfs；p.Asp615Asn | + | 1 | 0 |  |  |  |  |
|  |  | - | 13 | 0 |  |  |  |  |
|  | p.Asp45Argfs；p.Glu382* | + | 1 | 0 |  |  |  |  |
|  |  | - | 13 | 0 |  |  |  |  |
| VPS13D | p.G1190D;  p.Q1106X | + | 5 | 0 | 19 | 15 | 4 | Seong et al. (2018) |
|  |  | - | 10 | 4 |  |  |  |  |
|  | p.A4210V p.Y1803X | + | 2 | 0 |  |  |  |  |
|  |  | - | 13 | 4 |  |  |  |  |
|  | p.Gln662*  p.Met1307Leu | + | 1 | 0 |  |  |  |  |
|  |  | - | 14 | 4 |  |  |  |  |
|  | p.Gln2572* p.Gly4149Ser | + | 0 | 1 |  |  |  |  |
|  |  | - | 15 | 3 |  |  |  |  |
|  | p.Val2987Glyfs*14 c.941+3A>G | + | 1 | 0 |  |  |  |  |
|  |  | - | 14 | 4 |  |  |  |  |
|  | c.2237-1G>C p.Asn4107Ile | + | 1 | 0 |  |  |  |  |
|  |  | - | 14 | 4 |  |  |  |  |
|  | p.Leu2277* c.9998+4A>C | + | 0 | 1 |  |  |  |  |
|  |  | - | 15 | 3 |  |  |  |  |
|  | p.Val2445Glufs*16;  p.Asn3521Ser | + | 2 | 0 |  |  |  | Gauthier et al. (2018) |
|  |  | - | 13 | 4 |  |  |  |  |
|  | p.Arg4228Gln | + | 1 | 0 |  |  |  |  |
|  |  | - | 14 | 4 |  |  |  |  |
|  | c.5853-94_7148+210dup;  p.Leu2900Ser | + | 0 | 1 |  |  |  |  |
|  |  | - | 15 | 3 |  |  |  |  |
|  | p.Glu3573Aspfs*3;  p.Arg3253Gln | + | 1 | 1 |  |  |  |  |
|  |  | - | 14 | 3 |  |  |  |  |
|  | p.Thr865Ala;  p.Gly1200Asp | + | 1 | 0 |  |  |  |  |
|  |  | - | 14 | 4 |  |  |  |  |
